# Association of the *CHEK2* c.1100delC variant, radiotherapy, and systemic treatment with contralateral breast cancer risk and breast cancer‐specific survival

**DOI:** 10.1002/cam4.6272

**Published:** 2023-07-03

**Authors:** Anna Morra, Maartje A. C. Schreurs, Irene L. Andrulis, Hoda Anton‐Culver, Annelie Augustinsson, Matthias W. Beckmann, Sabine Behrens, Stig E. Bojesen, Manjeet K. Bolla, Hiltrud Brauch, Annegien Broeks, Saundra S. Buys, Nicola J. Camp, Jose E. Castelao, Melissa H. Cessna, Jenny Chang‐Claude, Wendy K. Chung, NBCS Collaborators, Kristine K. Sahlberg, Anne‐Lise Børresen‐Dale, Inger Torhild Gram, Karina Standahl Olsen, Olav Engebråten, Bjørn Naume, Jürgen Geisler, OSBREAC, Grethe I. Grenaker Alnæs, Sarah V. Colonna, Fergus J. Couch, Angela Cox, Simon S. Cross, Kamila Czene, Mary B. Daly, Joe Dennis, Peter Devilee, Thilo Dörk, Alison M. Dunning, Miriam Dwek, Douglas F. Easton, Diana M. Eccles, Mikael Eriksson, D. Gareth Evans, Peter A. Fasching, Tanja N. Fehm, Jonine D. Figueroa, Henrik Flyger, Marike Gabrielson, Manuela Gago‐Dominguez, Montserrat García‐Closas, José A. García‐Sáenz, Jeanine Genkinger, Felix Grassmann, Melanie Gündert, Eric Hahnen, Christopher A. Haiman, Ute Hamann, Patricia A. Harrington, Jaana M. Hartikainen, Reiner Hoppe, John L. Hopper, Richard S. Houlston, Anthony Howell, ABCTB Investigators, Christine Clarke, Deborah Marsh, Rodney Scott, Robert Baxter, Desmond Yip, Jane Carpenter, Alison Davis, Nirmala Pathmanathan, Peter Simpson, J. Dinny Graham, Mythily Sachchithananthan, kConFab Investigators, David Amor, Lesley Andrews, Yoland Antill, Rosemary Balleine, Jonathan Beesley, Ian Bennett, Michael Bogwitz, Leon Botes, Meagan Brennan, Melissa Brown, Michael Buckley, Jo Burke, Phyllis Butow, Liz Caldon, Ian Campbell, Michelle Cao, Anannya Chakrabarti, Deepa Chauhan, Manisha Chauhan, Georgia Chenevix‐Trench, Alice Christian, Paul Cohen, Alison Colley, Ashley Crook, James Cui, Eliza Courtney, Margaret Cummings, Sarah‐Jane Dawson, Anna DeFazio, Martin Delatycki, Rebecca Dickson, Joanne Dixon, Ted Edkins, Stacey Edwards, Gelareh Farshid, Andrew Fellows, Georgina Fenton, Michael Field, James Flanagan, Peter Fong, Laura Forrest, Stephen Fox, Juliet French, Michael Friedlander, Clara Gaff, Mike Gattas, Peter George, Sian Greening, Marion Harris, Stewart Hart, Nick Hayward, John Hopper, Cass Hoskins, Clare Hunt, Paul James, Mark Jenkins, Alexa Kidd, Judy Kirk, Jessica Koehler, James Kollias, Sunil Lakhani, Mitchell Lawrence, Jason Lee, Shuai Li, Geoff Lindeman, Lara Lipton, Liz Lobb, Sherene Loi, Graham Mann, Deborah Marsh, Sue Anne McLachlan, Bettina Meiser, Roger Milne, Sophie Nightingale, Shona O’Connell, Sarah O’Sullivan, David Gallego Ortega, Nick Pachter, Jia‐Min Pang, Gargi Pathak, Briony Patterson, Amy Pearn, Kelly Phillips, Ellen Pieper, Susan Ramus, Edwina Rickard, Bridget Robinson, Mona Saleh, Anita Skandarajah, Elizabeth Salisbury, Christobel Saunders, Jodi Saunus, Rodney Scott, Clare Scott, Adrienne Sexton, Andrew Shelling, Peter Simpson, Melissa Southey, Amanda Spurdle, Jessica Taylor, Renea Taylor, Heather Thorne, Alison Trainer, Kathy Tucker, Jane Visvader, Logan Walker, Rachael Williams, Ingrid Winship, Mary Ann Young, Milita Zaheed, Anna Jakubowska, Wolfgang Janni, Helena Jernström, Esther M. John, Nichola Johnson, Michael E. Jones, Vessela N. Kristensen, Allison W. Kurian, Diether Lambrechts, Loic Le Marchand, Annika Lindblom, Jan Lubiński, Michael P. Lux, Arto Mannermaa, Dimitrios Mavroudis, Anna Marie Mulligan, Taru A. Muranen, Heli Nevanlinna, Ines Nevelsteen, Patrick Neven, William G. Newman, Nadia Obi, Kenneth Offit, Andrew F. Olshan, Tjoung‐Won Park‐Simon, Alpa V. Patel, Paolo Peterlongo, Kelly‐Anne Phillips, Dijana Plaseska‐Karanfilska, Eric C. Polley, Nadege Presneau, Katri Pylkäs, Brigitte Rack, Paolo Radice, Muhammad U. Rashid, Valerie Rhenius, Mark Robson, Atocha Romero, Emmanouil Saloustros, Elinor J. Sawyer, Rita K. Schmutzler, Sabine Schuetze, Christopher Scott, Mitul Shah, Snezhana Smichkoska, Melissa C. Southey, William J. Tapper, Lauren R. Teras, Rob A. E. M. Tollenaar, Katarzyna Tomczyk, Ian Tomlinson, Melissa A. Troester, Celine M. Vachon, Elke M. van Veen, Qin Wang, Camilla Wendt, Hans Wildiers, Robert Winqvist, Argyrios Ziogas, Per Hall, Paul D. P. Pharoah, Muriel A. Adank, Antoinette Hollestelle, Marjanka K. Schmidt, Maartje J. Hooning

**Affiliations:** ^1^ Division of Molecular Pathology The Netherlands Cancer Institute Amsterdam the Netherlands; ^2^ Department of Medical Oncology Erasmus MC Cancer Institute Rotterdam the Netherlands; ^3^ Fred A. Litwin Center for Cancer Genetics Lunenfeld‐Tanenbaum Research Institute of Mount Sinai Hospital Toronto Ontario Canada; ^4^ Department of Molecular Genetics University of Toronto Toronto Ontario Canada; ^5^ Department of Medicine, Genetic Epidemiology Research Institute University of California Irvine Irvine California USA; ^6^ Oncology, Clinical Sciences in Lund Lund University Lund Sweden; ^7^ Department of Gynecology and Obstetrics, Comprehensive Cancer Center Erlangen‐EMN, Friedrich‐Alexander University Erlangen‐Nuremberg University Hospital Erlangen Erlangen Germany; ^8^ Division of Cancer Epidemiology German Cancer Research Center (DKFZ) Heidelberg Germany; ^9^ Copenhagen General Population Study, Herlev and Gentofte Hospital Copenhagen University Hospital Herlev Denmark; ^10^ Department of Clinical Biochemistry, Herlev and Gentofte Hospital Copenhagen University Hospital Herlev Denmark; ^11^ Faculty of Health and Medical Sciences University of Copenhagen Copenhagen Denmark; ^12^ Centre for Cancer Genetic Epidemiology, Department of Public Health and Primary Care University of Cambridge Cambridge UK; ^13^ Dr. Margarete Fischer‐Bosch‐Institute of Clinical Pharmacology Stuttgart Germany; ^14^ iFIT‐Cluster of Excellence University of Tübingen Tübingen Germany; ^15^ German Cancer Consortium (DKTK), Partner Site Tübingen German Cancer Research Center (DKFZ) Tübingen Germany; ^16^ Department of Internal Medicine and Huntsman Cancer Institute University of Utah Salt Lake City Utah USA; ^17^ Oncology and Genetics Unit, Instituto de Investigación Sanitaria Galicia Sur (IISGS) Xerencia de Xestion Integrada de Vigo‐SERGAS Vigo Spain; ^18^ Intermountain Healthcare Salt Lake City Utah USA; ^19^ Cancer Epidemiology Group, University Cancer Center Hamburg (UCCH) University Medical Center Hamburg‐Eppendorf Hamburg Germany; ^20^ Departments of Pediatrics and Medicine Columbia University New York New York USA; ^21^ Department of Cancer Genetics, Institute for Cancer Research Oslo University Hospital‐Radiumhospitalet Oslo Norway; ^22^ Faculty of Medicine, Institute of Clinical Medicine University of Oslo Oslo Norway; ^23^ Department of Research Vestre Viken Hospital Drammen Norway; ^24^ Department of Tumor Biology, Institute for Cancer Research Oslo University Hospital Oslo Norway; ^25^ Division of Surgery, Cancer and Transplantation Medicine, Department of Oncology Oslo University Hospital‐Radiumhospitalet Oslo Norway; ^26^ Department of Oncology Akershus University Hospital Lørenskog Norway; ^27^ Oslo Breast Cancer Research Consortium Oslo University Hospital Oslo Norway; ^28^ Department of Medical Genetics Oslo University Hospital and University of Oslo Oslo Norway; ^29^ Department of Community Medicine The Arctic University of Norway Tromsø Norway; ^30^ Core Facility for Biobanking The Arctic University of Norway Tromsø Norway; ^31^ Department of Laboratory Medicine and Pathology Mayo Clinic Rochester Minnesota USA; ^32^ Department of Oncology and Metabolism, Sheffield Institute for Nucleic Acids (SInFoNiA) University of Sheffield Sheffield UK; ^33^ Department of Neuroscience, Academic Unit of Pathology University of Sheffield Sheffield UK; ^34^ Department of Medical Epidemiology and Biostatistics Karolinska Institutet Stockholm Sweden; ^35^ Department of Clinical Genetics Fox Chase Cancer Center Philadelphia Pennsylvania USA; ^36^ Department of Pathology Leiden University Medical Center Leiden the Netherlands; ^37^ Department of Human Genetics Leiden University Medical Center Leiden the Netherlands; ^38^ Gynaecology Research Unit Hannover Medical School Hannover Germany; ^39^ Department of Oncology, Centre for Cancer Genetic Epidemiology University of Cambridge Cambridge UK; ^40^ School of Life Sciences University of Westminster London UK; ^41^ Faculty of Medicine University of Southampton Southampton UK; ^42^ Division of Evolution and Genomic Sciences, School of Biological Sciences, Faculty of Biology, Medicine and Health, University of Manchester Manchester Academic Health Science Centre Manchester UK; ^43^ North West Genomics Laboratory Hub, Manchester Centre for Genomic Medicine, St Mary's Hospital, Manchester University NHS Foundation Trust Manchester Academic Health Science Centre Manchester UK; ^44^ Department of Gynecology and Obstetrics, University Hospital Düsseldorf Heinrich‐Heine University Düsseldorf Düsseldorf Germany; ^45^ Usher Institute of Population Health Sciences and Informatics The University of Edinburgh Edinburgh UK; ^46^ Cancer Research UK Edinburgh Centre The University of Edinburgh Edinburgh UK; ^47^ Division of Cancer Epidemiology and Genetics, Department of Health and Human Services, National Cancer Institute National Institutes of Health Bethesda Maryland USA; ^48^ Department of Breast Surgery, Herlev and Gentofte Hospital Copenhagen University Hospital Herlev Denmark; ^49^ Cancer Genetics and Epidemiology Group, SERGAS, Instituto de Investigación Sanitaria de Santiago de Compostela (IDIS) Foundation Complejo Hospitalario Universitario de Santiago Santiago de Compostela Spain; ^50^ Medical Oncology Department, Hospital Clínico San Carlos, Instituto de Investigación Sanitaria San Carlos (IdISSC) Centro Investigación Biomédica en Red de Cáncer (CIBERONC) Madrid Spain; ^51^ Department of Epidemiology, Mailman School of Public Health Columbia University New York New York USA; ^52^ Herbert Irving Comprehensive Cancer Center New York New York USA; ^53^ Health and Medical University Potsdam Germany; ^54^ Molecular Epidemiology Group, C080 German Cancer Research Center (DKFZ) Heidelberg Germany; ^55^ Molecular Biology of Breast Cancer, University Womens Clinic Heidelberg University of Heidelberg Heidelberg Germany; ^56^ Institute of Diabetes Research, Helmholtz Zentrum München German Research Center for Environmental Health Neuherberg Germany; ^57^ Center for Familial Breast and Ovarian Cancer, Faculty of Medicine and University Hospital Cologne University of Cologne Cologne Germany; ^58^ Center for Integrated Oncology (CIO), Faculty of Medicine and University Hospital Cologne University of Cologne Cologne Germany; ^59^ Department of Preventive Medicine, Keck School of Medicine University of Southern California Los Angeles California USA; ^60^ Molecular Genetics of Breast Cancer German Cancer Research Center (DKFZ) Heidelberg Germany; ^61^ Translational Cancer Research Area University of Eastern Finland Kuopio Finland; ^62^ Institute of Clinical Medicine, Pathology and Forensic Medicine University of Eastern Finland Kuopio Finland; ^63^ University of Tübingen Tübingen Germany; ^64^ Centre for Epidemiology and Biostatistics, Melbourne School of Population and Global Health The University of Melbourne Melbourne Victoria Australia; ^65^ Division of Genetics and Epidemiology The Institute of Cancer Research London UK; ^66^ Division of Cancer Sciences University of Manchester Manchester UK; ^67^ Australian Breast Cancer Tissue Bank, Westmead Institute for Medical Research University of Sydney Sydney New South Wales Australia; ^68^ Research Department Peter MacCallum Cancer Center Melbourne Victoria Australia; ^69^ Sir Peter MacCallum Department of Oncology The University of Melbourne Melbourne Victoria Australia; ^70^ Department of Genetics and Pathology, International Hereditary Cancer Center Pomeranian Medical University Szczecin Poland; ^71^ Independent Laboratory of Molecular Biology and Genetic Diagnostics Pomeranian Medical University Szczecin Poland; ^72^ Department of Gynaecology and Obstetrics University Hospital Ulm Ulm Germany; ^73^ Department of Epidemiology and Population Health Stanford University School of Medicine Stanford California USA; ^74^ Division of Oncology, Department of Medicine, Stanford Cancer Institute Stanford University School of Medicine Stanford California USA; ^75^ The Breast Cancer Now Toby Robins Research Centre The Institute of Cancer Research London UK; ^76^ Laboratory for Translational Genetics, Department of Human Genetics KU Leuven Leuven Belgium; ^77^ VIB Center for Cancer Biology VIB Leuven Belgium; ^78^ Epidemiology Program University of Hawaii Cancer Center Honolulu Hawaii USA; ^79^ Department of Molecular Medicine and Surgery Karolinska Institutet Stockholm Sweden; ^80^ Department of Clinical Genetics Karolinska University Hospital Stockholm Sweden; ^81^ Biobank of Eastern Finland Kuopio University Hospital Kuopio Finland; ^82^ Department of Medical Oncology University Hospital of Heraklion Heraklion Greece; ^83^ Department of Laboratory Medicine and Pathobiology University of Toronto Toronto Ontario Canada; ^84^ Laboratory Medicine Program University Health Network Toronto Ontario Canada; ^85^ Department of Obstetrics and Gynecology, Helsinki University Hospital University of Helsinki Helsinki Finland; ^86^ Department of Oncology, Leuven Multidisciplinary Breast Center, University Hospitals Leuven Leuven Cancer Institute Leuven Belgium; ^87^ Institute for Medical Biometry and Epidemiology University Medical Center Hamburg‐Eppendorf Hamburg Germany; ^88^ Clinical Genetics Research Lab, Department of Cancer Biology and Genetics Memorial Sloan Kettering Cancer Center New York New York USA; ^89^ Clinical Genetics Service, Department of Medicine Memorial Sloan Kettering Cancer Center New York New York USA; ^90^ Department of Epidemiology, Gillings School of Global Public Health and UNC Lineberger Comprehensive Cancer Center University of North Carolina at Chapel Hill Chapel Hill North Carolina USA; ^91^ Department of Population Science American Cancer Society Atlanta Georgia USA; ^92^ IFOM ETS ‐ The AIRC Institute of Molecular Oncology, Genome Diagnostics Program Milan Italy; ^93^ Department of Medical Oncology Peter MacCallum Cancer Centre Melbourne Victoria Australia; ^94^ Sir Peter MacCallum Department of Oncology The University of Melbourne Parkville Victoria Australia; ^95^ Research Centre for Genetic Engineering and Biotechnology 'Georgi D. Efremov' MASA Skopje Republic of North Macedonia; ^96^ Division of Clinical Trials and Biostatistics, Department of Quantitative Health Sciences Mayo Clinic Rochester Minnesota USA; ^97^ Laboratory of Cancer Genetics and Tumor Biology, Cancer and Translational Medicine Research Unit, Biocenter Oulu University of Oulu Oulu Finland; ^98^ Laboratory of Cancer Genetics and Tumor Biology Northern Finland Laboratory Centre Oulu Oulu Finland; ^99^ Department of Experimental Oncology, Fondazione IRCCS Istituto Nazionale dei Tumori “Predictive Medicine: Molecular Bases of Genetic Risk” Milan Italy; ^100^ Department of Basic Sciences Shaukat Khanum Memorial Cancer Hospital and Research Centre (SKMCH & RC) Lahore Pakistan; ^101^ Medical Oncology Department Hospital Universitario Puerta de Hierro Madrid Spain; ^102^ Department of Oncology University Hospital of Larissa Larissa Greece; ^103^ School of Cancer & Pharmaceutical Sciences, Comprehensive Cancer Centre, Guy's Campus King's College London London UK; ^104^ Center for Molecular Medicine Cologne (CMMC), Faculty of Medicine and University Hospital Cologne University of Cologne Cologne Germany; ^105^ Medical Faculty, University Clinic of Radiotherapy and Oncology Ss. Cyril and Methodius University in Skopje Skopje Republic of North Macedonia; ^106^ Precision Medicine, School of Clinical Sciences at Monash Health Monash University Clayton Victoria Australia; ^107^ Department of Clinical Pathology The University of Melbourne Melbourne Victoria Australia; ^108^ Cancer Epidemiology Division Cancer Council Victoria Melbourne Victoria Australia; ^109^ Department of Surgery Leiden University Medical Center Leiden the Netherlands; ^110^ Cancer Research Centre The University of Edinburgh Edinburgh UK; ^111^ Division of Epidemiology, Department of Quantitative Health Sciences Mayo Clinic Rochester Minnesota USA; ^112^ Department of Clinical Science and Education, Södersjukhuset Karolinska Institutet Stockholm Sweden; ^113^ Department of Oncology Södersjukhuset Stockholm Sweden; ^114^ Family Cancer Clinic The Netherlands Cancer Institute ‐ Antoni van Leeuwenhoek Hospital Amsterdam the Netherlands; ^115^ Division of Psychosocial Research and Epidemiology The Netherlands Cancer Institute ‐ Antoni van Leeuwenhoek Hospital Amsterdam the Netherlands

**Keywords:** *CHEK2* c.1100delC germline genetic variant, contralateral breast cancer risk, radiotherapy, survival, systemic treatment

## Abstract

**Background:**

Breast cancer (BC) patients with a germline CHEK2 c.1100delC variant have an increased risk of contralateral BC (CBC) and worse BC‐specific survival (BCSS) compared to non‐carriers.

**Aim:**

To assessed the associations of CHEK2 c.1100delC, radiotherapy, and systemic treatment with CBC risk and BCSS.

**Methods:**

Analyses were based on 82,701 women diagnosed with a first primary invasive BC including 963 CHEK2 c.1100delC carriers; median follow‐up was 9.1 years. Differential associations with treatment by CHEK2 c.1100delC status were tested by including interaction terms in a multivariable Cox regression model. A multi‐state model was used for further insight into the relation between CHEK2 c.1100delC status, treatment, CBC risk and death.

**Results:**

There was no evidence for differential associations of therapy with CBC risk by CHEK2 c.1100delC status. The strongest association with reduced CBC risk was observed for the combination of chemotherapy and endocrine therapy [HR (95% CI): 0.66 (0.55–0.78)]. No association was observed with radiotherapy. Results from the multi‐state model showed shorter BCSS for CHEK2 c.1100delC carriers versus non‐carriers also after accounting for CBC occurrence [HR (95% CI): 1.30 (1.09–1.56)].

**Conclusion:**

Systemic therapy was associated with reduced CBC risk irrespective of CHEK2 c.1100delC status. Moreover, CHEK2 c.1100delC carriers had shorter BCSS, which appears not to be fully explained by their CBC risk.

## 1 INTRODUCTION

Breast cancer (BC) has the highest incidence in women worldwide.^1^ One of the germline variants that confer a moderate increased BC risk is the *CHEK2* c.1100delC variant,^2^, ^3^, ^4^ which is found in approximately 0.7% of the Northern and Western European populations.^5^ Overall, carriers of this variant are diagnosed at a younger age than non‐carriers^4^ and the majority develops BCs that are estrogen receptor (ER)‐ and progesterone receptor (PR)‐positive and human epidermal growth factor receptor 2 (HER2)‐negative.^3^, ^6^ Although this BC subtype has the most favorable prognosis in the general BC population,^7^ *CHEK2* c.1100delC carriers have a higher risk of developing contralateral breast cancer (CBC) and worse survival^3^, ^4^, ^6^, ^8^, ^9^ compared to non‐carriers.

Reasons behind these differences are still unclear. A possible explanation is that *CHEK2* c.1100delC carriers have a different response to treatment compared to non‐carriers. Radiotherapy has been shown to increase the risk of CBC in the general BC population, especially in younger patients.^10^ Treatment with radiotherapy causes DNA strand breaks, which are less likely to be repaired in *CHEK2* c.1100delC carriers.^11^ While this might be beneficial for the treatment of the first primary cancer, carriers might be more prone to developing a CBC.^12^ One case‐only study showed a non‐significant increased risk for developing CBC after treatment with radiotherapy in *CHEK2* c.1100delC carriers versus non‐carriers, but due to the small study size, the associations in the younger population could not be investigated.^13^ Only one other small study reported on the association between radiotherapy and CBC risk by *CHEK2* c.1100delC status.^8^

On the other hand, less is known about whether the impact of systemic therapy on CBC risk and survival differs by CHEK2 c.1100delC status. A population‐based study showed a significant decrease in CBC risk following chemotherapy and endocrine therapy in BC overall.^14^ One single‐hospital study also found a decreased risk of CBC after chemotherapy use in *CHEK2* c.1100delC carriers, and did not find evidence for a differential association by *CHEK2* c.1100delC status.^15^ That study also found no evidence for a differential impact of chemotherapy on survival.^15^

Given this uncertainty, our aim was to assess, within a large international cohort, potential differential associations of treatment given for the first primary BC (i.e., radiotherapy, chemotherapy, and endocrine therapy) by *CHEK2* c.1100delC status with CBC risk and to investigate whether the worse breast cancer‐specific survival (BCSS) so far reported in carriers is explained solely by the increased CBC risk.

## 2 MATERIALS AND METHODS

### 2.1 Study population

**FIGURE 1:**
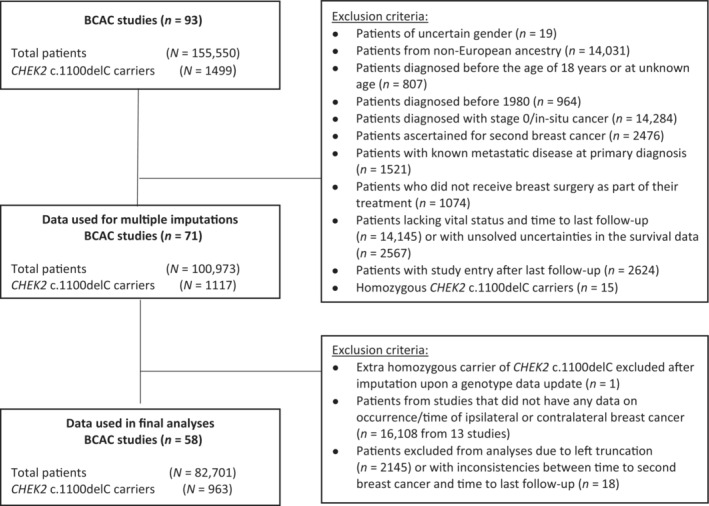
Data flowchart of inclusion and exclusion of patients with breast cancer from the Breast Cancer Association Consortium (BCAC) database.

We used data from the Breast Cancer Association Consortium (BCAC), selected women of European ancestry, diagnosed with a first primary invasive BC between 1980 and 2018; exclusion criteria are shown in Figure 1. The main analyses were based on 82,701 BC patients from 58 BCAC studies (Table S1). All individual studies were approved by the appropriate institutional review boards and/or medical ethical committees. Written informed consent was obtained from all study participants.

Previous analyses investigating the relationship between *CHEK2* c.1100delC status, risk of CBC, and mortality have been based on a subset of patients genotyped with Taqman.^3^, ^4^ In particular, the current study includes most carriers from the Weischer et al. study (*n* = 459)^4^ and from the Kriege et al. study (*n* = 193),^15^ but is based on a larger number of BC patients and includes updated follow‐up data.

### 2.2 Data collection

All relevant clinical‐pathological and treatment information, as well as outcome information, was collected by individual studies and harmonized by the BCAC Survival, Pathology and Treatment Working Group at the Netherlands Cancer Institute, Amsterdam, the Netherlands, in collaboration with the individual studies before incorporation into the BCAC database (version 13, May 2021). *CHEK2* c.1100delC status was obtained from five different sources: BRIDGES sequencing data,^16^ Taqman and iPLEX genotyping^3^, ^4^, ^17^ (56.5% of the included study individuals: 0.9% *CHEK2* c.1100delC carriers and 55.6% non‐carriers, respectively), and imputed genotypes from OncoArray^18^ (32.0% of the included study individuals: 0.02% defined as CHEK2 c.1100delC carriers and 31.8% defined as non‐carriers based on imputed dosages) or iCOGS^19^ (the remaining 11.5% of the included study individuals: 0.1% defined as *CHEK2* c.1100delC carriers and 11.4% as non‐carriers based on imputed dosages) as described in the Supplementary Methods.

### 2.3 Statistical analyses

Multiple imputation, performed using R package MICE (version 3.13.0), was used to handle missing values in clinical and pathological variables. Details are given in the Supplementary Methods and Table S2. Descriptive statistics are shown as mean ± standard deviation (SD) or median and interquartile range (IQR). We used Pearson's chi‐squared test for categorical data and Kruskal–Wallis test for continuous data to calculate differences in patients' characteristics. The primary study outcomes were time to CBC and BCSS (time to death due to BC).

Hazard ratios (HRs) and 95% confidence intervals (CIs) for the association of treatment given for the first primary BC (radiotherapy and/or type of systemic treatment) and *CHEK2* c.1100delC status with time to CBC were estimated via Cox regression models allowing for delayed entry, stratified by country and adjusted for age at first primary BC diagnosis, tumor size, nodal status, grade, and ER status. Since ER status is known to violate the proportionality hazards assumption and because the majority of *CHEK2* c.1100delC carriers develop ER‐positive BC, we performed an additional main analysis restricted to patients diagnosed with a first primary ER‐positive BC. We assumed that patients with unknown CBC status did not develop a CBC during follow‐up and that for CBC cases with unknown time from first primary BC to CBC diagnosis, CBC occurrence was at last available follow‐up.

Time at risk started either 3 months after first primary BC diagnosis or at study entry if entry was more than 3 months after first primary BC diagnosis, and ended at time of CBC, death or last follow‐up, whichever came first. We tested for potential differential association of adjuvant and/or neo‐adjuvant therapy on CBC risk according to *CHEK2* c.1100delC status by including an interaction term between treatment (radiotherapy or systemic treatment) variable and *CHEK2* c.1100delC status in the model. CBC risk analyses were stratified by two follow‐up time intervals: (i) the first 5 years after BC diagnosis and (ii) starting 5 years after BC diagnosis.

**FIGURE 2:**
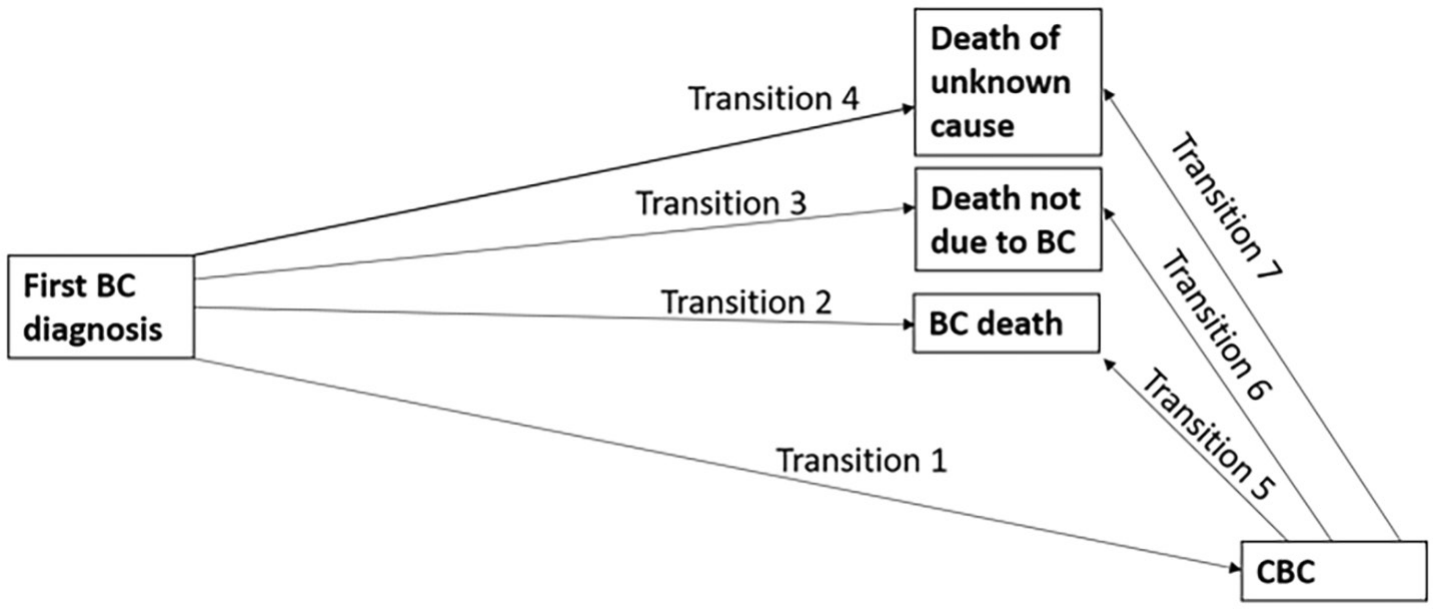
Graphical representation of the multi‐state model. BC, breast cancer; CBC, contralateral breast cancer.

To gain further insight into the relation between *CHEK2* c.1100delC status, treatment given for the first primary BC, CBC risk, and death, we used a multi‐state model in the framework of the Cox model, with diagnosis of the first primary BC as initial state, diagnosis of CBC as intermediate (transient) state, and death due to BC, death due to other causes, and death due to unknown causes as absorbing states (Figure 2), as specified in the Supplementary Methods.

The main CBC risk and multi‐state analyses were performed on imputed datasets. Complete‐case analyses (excluding study subjects with missing values in any of the variables included in the models) were performed as sensitivity analyses. Additional analyses were restricted to: (a) patients diagnosed with first primary BC from 2000 onwards to reduce heterogeneity in treatment regimens; (b) patients diagnosed at age 40 or younger to see if the association with radiotherapy was stronger in this subgroup, as reported previously in the general BC population.^10^

## 3 RESULTS

This study included data from 963 *CHEK2* c.1100delC carriers and 81,738 non‐carriers. Patients carrying the *CHEK2* c.1100delC variant were diagnosed with a first primary invasive BC at a younger age (median age 52 years in carriers compared to 56 years in non‐carriers) and in earlier calendar years (36.4% of carriers was diagnosed before 2000, compared to 27.6% of the non‐carriers). The tumors of carriers were larger at time of diagnosis and were more often lymph node‐positive, grade 2, and ER‐ and PR‐positive than in non‐carriers. Furthermore, carriers more often underwent a mastectomy as part of their treatment compared to non‐carriers and more often did not receive any systemic therapy compared to the non‐carriers (Table 1).

**TABLE 1:** Clinical, tumor and treatment characteristics for the first primary BC by *CHEK2* c.1100delC carrier status.

Characteristics	Non‐carriers	*CHEK2* c.1100delC carriers	*p*‐value
Number of patients, *n*	81,738	963	
Number of patients diagnosed with CBC, *n* (%)	1757 (2.1)	59 (6.1)	
Number of patients diagnosed with ipsilateral BC, *n* (%)^a^	517 (0.6)	6 (0.6)	
Total FU time, years (IQR)	9.2 (5.3–13.6)	9.6 (5.5–13.9)	
*Clinical risk factors*			
Age at diagnosis, *y*, median (IQR)	56 (47–64)	52 (44–61)	<0.001
Age at diagnosis, *n* (%)			<0.001
<40 years	9471 (11.6)	171 (17.8)	
40–50 years	19,978 (24.4)	277 (28.8)	
50–60 years	23,044 (28.2)	266 (27.6)	
>60 years	29,245 (35.8)	249 (25.9)	
Year of diagnosis, *n* (%)			<0.001
1980–1989	2259 (2.8)	48 (5.1)	
1990–1999	20,055 (24.8)	297 (31.3)	
2000–2009	45,910 (56.7)	492 (51.8)	
≥2010	12,781 (15.8)	113 (11.9)	
Missing, *n*	733	13	
*Tumor characteristics*			
Tumor size, *n* (%)			0.01
≤2 cm	40,263 (63.0)	421 (58.6)	
>2 and ≤5 cm	20,977 (32.8)	273 (38.0)	
>5 cm	2718 (4.3)	24 (3.3)	
Missing, *n*	17,780	245	
Lymph node status, *n* (%)			<0.001
Negative	42,079 (61.4)	439 (54.8)	
Positive	26,456 (38.6)	362 (45.2)	
Missing, *n*	13,203	162	
Grade, *n* (%)			0.01
Grade 1	12,572 (19.1)	112 (15.3)	
Grade 2	31,594 (48.1)	388 (53.0)	
Grade 3	21,536 (32.8)	232 (31.7)	
Missing, *n*	16,036	231	
Morphology, *n* (%)			0.16
Ductal	52,127 (74.0)	659 (77.5)	
Lobular	10,596 (15.0)	116 (13.7)	
Medullary	619 (0.9)	3 (0.4)	
Mixed (ductal and lobular)	3032 (4.3)	37 (4.4)	
Mucinous	895 (1.3)	7 (0.8)	
Papillary	160 (0.2)	22 (0.1)	
Tubular	908 (1.3)	1 (0.6)	
Other	2111 (3.0)	5 (2.6)	
Missing, *n*	11,290	113	
ER status, *n* (%)			<0.001
Negative	13,918 (20.4)	93 (11.8)	
Positive	54,481 (79.7)	694 (88.2)	
Missing, *n*	13,339	176	
PR status, *n* (%)			<0.001
Negative	19,128 (32.1)	169 (24.5)	
Positive	40,548 (68.0)	520 (75.5)	
Missing, *n*	22,062	274	
HER2 status, *n* (%)			0.55
Negative	37,395 (83.5)	418 (82.5)	
Positive	7376 (16.5)	89 (17.6)	
Missing, *n*	36,967	456	
*Treatment*			
Surgery, *n* (%)			<0.001
Breast conserving surgery	23,706 (43.3)	244 (36.3)	
Mastectomy	16,129 (29.4)	259 (38.5)	
Type unknown	15,330 (27.6)	169 (25.2)	
Missing, *n*	26,573	291	
Radiotherapy, *n* (%)			0.36
No	13,163 (26.0)	181 (27.6)	
Yes	37,479 (74.0)	474 (72.4)	
Missing, *n*	31,096	308	
Systemic therapy, *n* (%)			<0.001
No systemic therapy	4996 (11.2)	94 (17.0)	
CT, no ET	7501 (16.8)	88 (15.9)	
ET, no CT	16,976 (38.1)	153 (27.7)	
Both CT and ET	15,116 (33.9)	218 (39.4)	
Missing, *n*	37,149	410	
Trastuzumab, *n* (%)			0.96
No	37,466 (95.4)	478 (95.2)	
Yes	1819 (4.6)	24 (4.8)	
Missing, *n*	42,453	461	

*Note*: Percentages are only on observed, non‐missing data, and may not total 100 because of rounding.

Abbreviations: CBC, contralateral breast cancer; CT, chemotherapy; ER, estrogen receptor; ET, endocrine therapy; HER2, human epidermal growth factor receptor 2; PR, progesterone receptor.

^a^ Data component not actively collected in BCAC.

### 3.1 Contralateral breast cancer

*CHEK2* c.1100delC carriers were diagnosed with CBC at younger age and in earlier calendar years. Overall, the characteristics of the CBC were similar between the non‐carriers and carriers (Table S3). However, *CHEK2* c.1100delC carriers more often had positive nodes at CBC diagnosis than non‐carriers (*p* = 0.02).

### 3.2 Contralateral breast cancer risk by treatment and *CHEK2* c.1100delC carrier status

There was no evidence for a differential association of *CHEK2* c.1100delC status by radiotherapy (Tables 2 and 3: *p*‐value for interaction = 0.31 in all patients and *p*‐value for interaction = 0.99 in ER‐positive patients) or systemic therapy (*p*‐value for interaction = 0.46 in all patients and *p*‐value for interaction = 0.68 in ER‐positive patients). Moreover, we did not find an association of radiotherapy with CBC risk [HR (95% CI): 1.07 (0.94–1.21), *p* = 0.33 in all BC patients and 1.07 (0.92–1.25), *p* = 0.35 in ER‐positive BC patients]. Regarding systemic therapy, we observed that chemotherapy alone [HR (95% CI): 0.77 (0.62–0.96), *p* = 0.02 in all BC patients and 0.73 (0.52–1.03), *p* = 0.07 in ER‐positive BC patients], endocrine therapy alone [HR (95% CI): 0.70 (0.58–0.83), *p* < 0.001 in all BC patients and 0.66 (0.54–0.81), *p* < 0.001 in ER‐positive BC patients], and the combination of both [HR (95% CI): 0.65 (0.55–0.78), *p* < 0.001 in all BC patients and 0.65 (0.52–0.82), *p* < 0.001 in ER‐positive BC patients] were associated with lower CBC risk compared to women who did not receive any systemic therapy as part of their treatment.

**TABLE 2:** Contralateral breast cancer risk (hazard ratio) by treatment for first primary breast cancer and *CHEK2* c.1100delC status. Stratified by time since first primary breast cancer diagnosis.

	Total follow‐up time			<5‐year follow‐up			>5 years follow‐up		
No of patients	82,701			73,354			62,688		
No of CBC events	1816			656			1160		
	HR (95% CI)	*p*‐value	*p*‐int	HR (95% CI)	*p*‐value	*p*‐int	HR (95% CI)	*p*‐value	*p*‐int
*CHEK2* c.1100delC status	2.37 (1.82–3.08)	<0.001		3.08 (2.12–4.48)	<0.001		1.93 (1.33–2.80)	<0.001	
Radiotherapy			0.31			0.30			0.77
No radiotherapy	Ref			Ref			Ref		
Radiotherapy	1.07 (0.94–1.21)	0.33		0.98 (0.81–1.19)	0.84		1.12 (0.96–1.31)	0.16	
Systemic therapy			0.46			0.70			0.39
No systemic therapy	Ref			Ref			Ref		
CT, no ET	0.77 (0.62–0.96)	0.02		0.58 (0.41–0.83)	0.003		0.90 (0.70–1.15)	0.39	
ET, no CT	0.70 (0.58–0.83)	<0.001		0.62 (0.46–0.84)	0.002		0.73 (0.59–0.91)	0.005	
Both CT and ET	0.65 (0.55–0.78)	<0.001		0.50 (0.37–0.68)	<0.001		0.75 (0.62–0.93)	0.007	

*Note*: Adjusted for age at diagnosis, ER status, tumor size, nodal status and grade of first primary breast cancer.

Abbreviations: CBC, contralateral breast cancer; CT, chemotherapy; ER, estrogen receptor; ET, endocrine therapy; *p*‐int, *p*‐value for the comparison of a model including an interaction term between *CHEK2* c.1100delC status and a specific treatment (radiation or systemic treatment) with a model without any interaction term.

**TABLE 3:** Contralateral breast cancer risk (hazard ratio) by treatment for first primary BC and *CHEK2* c.1100delC status in ER‐positive BC patients. Stratified by time since first primary breast cancer diagnosis.

	Total follow‐up time			<5‐year follow‐up			>5 years follow‐up		
No. of patients	55,175			51,146			41,269		
No. of CBC events	1133			427			706		
	HR (95% CI)	*p*‐value	*p*‐int	HR (95% CI)	*p*‐value	*p*‐int	HR (95% CI)	*p*‐value	*p*‐int
*CHEK2* c.1100delC status	2.55 (1.87–3.48)	<0.001		3.42 (2.24–5.22)	<0.001		1.94 (1.22–3.08)	0.005	
Radiotherapy			0.99			0.47			0.41
No radiotherapy	Ref			Ref			Ref		
Radiotherapy	1.07 (0.92–1.25)	0.35		1.04 (0.81–1.34)	0.75		1.09 (0.90–1.32)	0.36	
Systemic therapy			0.68			0.91			0.96
No systemic therapy	Ref			Ref			Ref		
CT, no ET	0.73 (0.52–1.03)	0.07		0.62 (0.38–1.03)	0.06		0.80 (0.52–1.23)	0.31	
ET, no CT	0.66 (0.54–0.81)	<0.001		0.55 (0.40–0.77)	<0.001		0.73 (0.57–0.94)	0.02	
Both CT and ET	0.65 (0.52–0.82)	<0.001		0.48 (0.34–0.69)	<0.001		0.77 (0.58–1.03)	0.08	

*Note*: Adjusted for age at diagnosis, nodal status, size category and grade of first primary breast cancer.

Abbreviations: CT, chemotherapy; ER, estrogen receptor; ET, endocrine therapy; *p*‐int, *p*‐value for the comparison of a model including an interaction term between *CHEK2* c.1100delC status and a specific treatment (radiotherapy or systemic treatment) with a model without any interaction term.

Results of analyses for patients diagnosed at the age of 40 years or younger or for patients diagnosed from 2000 onwards were in line with the results of the main analyses (Tables S4 and S5). Complete‐case analyses results were consistent with the corresponding results of the imputed data analyses (Tables S6–S9), except for the association with radiotherapy in patients diagnosed at the age of 40 years or younger. For these patients, radiotherapy was significantly associated with increased CBC risk in the complete‐case analysis with follow‐up starting 5 years after diagnosis of the first primary BC [Table S7; HR (95% CI): 2.12 (1.06–4.22), *p* = 0.03]. In addition, interaction terms between treatments and *CHEK2* c.1100delC status could not be properly estimated in some of the complete‐case analyses, due to insufficient data. These included, among others, the analysis based on all patients with follow‐up starting at 5 years after BC diagnosis; the analysis restricted to patients diagnosed at the age of 40 years or younger and based on the total follow‐up; and the analysis restricted to ER‐positive BC with follow‐up starting 5 years after BC diagnosis (Tables S10–S12).

### 3.3 *CHEK2* c.1100delC carrier status, contralateral breast cancer, and survival trajectories

*CHEK2* c.1100delC carriers versus non‐carriers had an almost 2.4 fold risk of developing a CBC [HR (95% CI): 2.37 (1.82–3.08), *p* < 0.001 in all patients and 2.55 (1.87–3.48), *p* < 0.001 in patients with an ER‐positive first primary BC; Table 4] and a 1.3‐fold risk of BC death after censoring for CBC occurrence [HR (95% CI): 1.30 (1.09–1.56), *p* = 0.003 in all patients and 1.38 (1.12–1.71), *p* = 0.003 in patients with an ER‐positive first primary BC; Table 4]. There was no evidence for association of *CHEK2* c.1100delC carrier status with other transitions. Results from the analyses restricted to patients diagnosed with first primary BC from 2000 onwards were in line with the results from the main analyses (Table S15).

**TABLE 4:** Multi‐state model in all breast cancer patients and in patients diagnosed with a first primary ER‐positive breast cancer: hazard ratio for the comparison of *CHEK2* c.1100delC carriers versus non‐carriers for each transition.

Analysis	Transition	Description	HR (95% CI)	*p*	Cases	Events
All BC patients	1	First primary BC → CBC	2.37 (1.82–3.08)	<0.001	82,701	1816
	2	First primary BC → BC death	1.30 (1.09–1.56)	0.003		7467
	3	First primary BC → death not due to BC	1.00 (0.75–1.34)	0.98		4247
	4	First primary BC → death of unknown cause	1.07 (0.76–1.49)	0.70		3548
	5	CBC → BC death	1.23 (0.72–2.10)	0.46	1816	281
	6	CBC → death not due to BC	0.60 (0.14–2.52)	0.49		124
	7	CBC → death of unknown cause	1.21 (0.41–3.53)	0.73		94
Patients diagnosed with primary ER‐positive BC	1	First primary BC → CBC	2.55 (1.87–3.48)	<0.001	55,175	1133
	2	First primary BC → BC death	1.38 (1.12–1.71)	0.003		4266
	3	First primary BC → death not due to BC	1.13 (0.81–1.56)	0.47		2817
	4	First primary BC → death of unknown cause	0.97 (0.63–1.48)	0.87		2090
	5	CBC → BC death	1.49 (0.79–2.81)	0.21	1133	167
	6	CBC → death not due to BC	0.89 (0.20–4.06)	0.89		80
	7	CBC → death of unknown cause	0.61 (0.14–2.79)	0.53		55

*Note*: The models included age at first primary BC diagnosis, nodal status, tumor size, grade, radiotherapy and systemic treatment given for the first primary BC as covariates. The model based on all BC patient included ER status of the first primary BC as additional covariate. Baseline hazards were allowed to vary across country and transition. All the estimates from the model are shown in Tables S13 and S14.

Abbreviations: BC, breast cancer; CBC, contralateral breast cancer; CI, confidence interval; ER, estrogen receptor; HR, hazard ratio.

Regarding treatment, radiotherapy was associated with lower risk of death due to causes other than BC or unknown causes, while there was no significant association with BC‐specific death (Tables S13–S15). Endocrine therapy alone was associated with a significantly decreased risk of BC‐specific death (particularly in patients diagnosed with an ER‐positive first primary BC) and with a highly significantly decreased risk of death due to unknown causes. The combination of endocrine therapy and chemotherapy was associated with decreased risk of BC death (in patients diagnosed with an ER‐positive first primary BC), with risk of death due to causes other than BC and had the strongest protective association against death due to unknown causes (Table S14). The corresponding complete‐case analyses showed similar patterns of association (Tables S16–S18).

## 4 DISCUSSION

The main goal of this study was to assess potential differential associations of treatment by *CHEK2* c.1100delC status with CBC risk and to investigate if the poorer survival in *CHEK2* c.1100delC carriers may be explained alone by the occurrence of CBC. The Breast Cancer Association Consortium provided a unique resource of 963 carriers of this single *CHEK2* variant to study this question in more detail.

These data did not support the hypothesis of differential associations of treatment with CBC risk by *CHEK2* c.1100delC status. As expected, systemic therapy was found to decrease CBC risk, with the strongest association in the first 5 years after first primary BC diagnosis, when endocrine therapy is likely to be ongoing.^14^, ^20^ Overall, we did find that the combination of endocrine therapy with chemotherapy resulted in the largest reduction in CBC risk, which has been previously reported.^14^ The lack of evidence for a differential association of systemic therapy with CBC risk by *CHEK2* c.1100delC status suggests that carriers experience a similar beneficial effect as non‐carriers. This is in line with previous studies in *CHEK2* c.1100delC carriers.^15^, ^21^, ^22^

In addition, we did not find a significant association of radiotherapy with CBC risk. This lack of association is in contrast with previous studies in sporadic BC patients, which showed that radiotherapy is a contributor to CBC risk, especially when treatment was administered at a younger age.^10^, ^23^, ^24^, ^25^ One explanation for this might be the change of radiation techniques over time. However, analyses restricted to patients diagnosed from the year 2000 onwards, when treatment regimens were expected to be more homogeneous, showed similar results as were found in the main analyses. Therefore, although observational—and non‐randomized—studies like the present cannot rebut hypotheses of causality, these changes are unlikely to be the reason behind the lack of association between radiotherapy and CBC risk in our study.

In line with previous studies,^3^, ^4^ we found a greater than twofold increased risk of CBC in *CHEK2* c.1100delC carriers compared to non‐carriers. This is consistent with the reported increase in risk of a first primary BC,^2^, ^16^ suggesting that genetic variants that predispose to the development of a first primary BC will also predispose to the development of a CBC. We also observed a shorter BCSS in *CHEK2* c.1100delC carriers compared to non‐carriers, after accounting for CBC occurrence, age at diagnosis of the first primary BC and tumor characteristics. This suggests that the shorter BCSS in *CHEK2* c.1100delC carriers versus non‐carriers is partly explained by a component other than the established prognostic factors. Moreover, *CHEK2* c.1100delC carriers were on average diagnosed in earlier calendar years compared to non‐carriers. Therefore, carriers probably received less efficacious chemotherapy and endocrine therapy compared to non‐carriers, which could have affected survival.

The main strengths of our study are the large sample size, including information about tumor pathology, treatment, time to CBC and survival, and a median follow‐up of over 9 years. In addition, the use of a multi‐state model provides important advantages compared to individual survival models with different endpoints. By modeling all events of interest together, the multi‐state model gives insight on how intermediate events, such as CBC, affect survival. Moreover, it allows estimation of associations with transition‐specific treatment and covariates, thereby providing insight on whether and to what extent the associations change across transitions and corresponding endpoints. Most of the studies were hospital‐ or population‐based, and most BC patients were unaware of a *CHEK2* variant, which we determined in the research setting. Therefore, it is highly unlikely that knowledge of carrier status could have affected clinical data collection.

There are some limitations to our study that need to be acknowledged. Between studies there was minor heterogeneity in the definition of stage, grade, and cut‐offs for ER, PR, and HER2 status, which would have affected both carriers and non‐carriers to a similar extent and is unlikely to have impacted our conclusions. Many of the variables related to tumor characteristics and treatment had large proportions of missing values. Complete‐case analyses have less power to detect the associations of interest and might be biased if case data are not missing completely at random.^26^ We addressed the missing data problem by employing multiple imputation,^26^ which should provide unbiased estimates, assuming that data are missing at random and that imputation models are correctly specified. Analyses restricted to complete‐case data yielded results that were mostly consistent with the results based on imputed data. In addition, in some complete‐case analyses, the number of *CHEK2* c.1100delC carriers was too low to properly estimate the interaction terms. This underlines the importance of the analyses based on imputed data, which avoids losses in the number of cases and events in the analyses. We also did not consider type of chemotherapy or endocrine therapy in the analyses, nor had we information about ovarian function suppression. Moreover, information about the occurrence of primary ipsilateral BCs was very limited and could not be properly accounted for in our analyses. However, based on the available information, there was no difference in the proportion of ipsilateral BC between *CHEK2* c.1100delC carriers and non‐carriers (0.6% in both groups) and is unlikely to have had a major impact on our BCSS results. An additional limitation was the lack of information on cause of death for about 25% of those who had died. This would result in a loss of power to detect associations with BCSS if most of the deaths of unknown causes were due to BC. However, this would, at worst, dilute our results rather than leading to false‐positive significant associations with BCSS. Finally, while we accounted for several established BC prognostic factors in our analyses, we cannot exclude the presence of residual bias affecting to some extent our results. An example of such bias is known as “indication bias,” which applies to the presence of an indication which causes or affects the outcome of interest.^27^ This could explain some of the unexpected results for the association of radiotherapy and systemic treatment with death‐related outcomes, in case treatment decisions are influenced by the presence/absence of certain conditions or morbidities in such a way that patients receiving the treatment are less likely to die from other causes than BC. While indication bias could have affected the treatment‐related effects on mortality, it is less likely to be an issue for the association of *CHEK2* c.1100delC status and treatment with CBC risk and survival.

In conclusion, the results of our study did not provide evidence for differential associations with radiation or systemic therapy by *CHEK2* c.100delC status on CBC risk. This suggests that associations of these treatments with CBC risk are similar between carriers and non‐carriers. Furthermore, we confirmed the presence of a risk component for BC‐specific death in *CHEK2* c.1100delC carriers which is not explained by CBC occurrence or characteristics of the first primary BC. Genotyping of *CHEK2* c.1100delC in patients of ongoing clinical trials would allow the evaluation of treatment response in detail and determine any impact of the *CHEK2* c.1100delC variant on the efficacy of BC treatment. In addition, studies focusing on, for example, the molecular copy number aberration profile of *CHEK2*‐related tumors should further shed light on potential biological mechanisms underlying the observed increased CBC risk and possible worse survival in CHEK2 c.1100delC carriers.

## AUTHOR CONTRIBUTIONS

**Anna Morra:** Conceptualization (equal); data curation (equal); formal analysis (equal); investigation (equal); methodology (equal); software (equal); writing – original draft (equal); writing – review and editing (equal). **Maartje A. C. Schreurs:** Conceptualization (equal); data curation (equal); formal analysis (equal); investigation (equal); methodology (equal); software (equal); writing – original draft (equal); writing – review and editing (equal). **Irene L. Andrulis:** Funding acquisition (equal); resources (equal); writing – review and editing (equal). **Hoda Anton‐Culver:** Funding acquisition (equal); resources (equal); writing – review and editing (equal). **Annelie Augustinsson:** Funding acquisition (equal); resources (equal); writing – review and editing (equal). **Mattias W Beckman:** Funding acquisition (equal); resources (equal); writing – review and editing (equal). **Sabine Behrens:** Data curation (equal); funding acquisition (equal); resources (equal); writing – review and editing (equal). **Stig E Bojesen:** Funding acquisition (equal); resources (equal); writing – review and editing (equal). **Manjeet K. Bolla:** Data curation (equal); funding acquisition (equal); resources (equal); writing – review and editing (equal). **Hiltrud Brauch:** Funding acquisition (equal); resources (equal); writing – review and editing (equal). **Annigien Broeks:** Funding acquisition (equal); resources (equal); writing – review and editing (equal). **Saundra S. Buys:** Funding acquisition (equal); resources (equal); writing – review and editing (equal). **Nicola J. Camp:** Funding acquisition (equal); resources (equal); writing – review and editing (equal). **Jose E. Castelao:** Funding acquisition (equal); resources (equal); writing – review and editing (equal). **Melissa H. Cessna:** Funding acquisition (equal); resources (equal); writing – review and editing (equal). **Jenny Chang‐Claude:** Funding acquisition (equal); resources (equal); writing – review and editing (equal). **Wendy Chung:** Funding acquisition (equal); resources (equal); writing – review and editing (equal). **NBCS Collaborators:** Funding acquisition (equal); resources (equal); writing – review and editing (equal). **Sarah V. Colonna:** Funding acquisition (equal); resources (equal); writing – review and editing (equal). **Fergus J. Couch:** Funding acquisition (equal); resources (equal); writing – review and editing (equal). **Angela Cox:** Funding acquisition (equal); resources (equal); writing – review and editing (equal). **Simon S. Cross:** Funding acquisition (equal); resources (equal); writing – review and editing (equal). **Kamila Czene:** Funding acquisition (equal); resources (equal); writing – review and editing (equal). **Mary B. Daly:** Funding acquisition (equal); resources (equal); writing – review and editing (equal). **Joe Dennis:** Funding acquisition (equal); resources (equal); writing – review and editing (equal). **Peter Devilee:** Funding acquisition (equal); resources (equal); writing – review and editing (equal). **Thilo Dörk:** Funding acquisition (equal); resources (equal); writing – review and editing (equal). **Alison M. Dunning:** Funding acquisition (equal); resources (equal); writing – review and editing (equal). **Miriam V Dwek:** Funding acquisition (equal); resources (equal); writing – review and editing (equal). **Douglas F. Easton:** Funding acquisition (equal); resources (equal); writing – review and editing (equal). **Diana Eccles:** Funding acquisition (equal); resources (equal); writing – review and editing (equal). **Mikael Eriksson:** Funding acquisition (equal); resources (equal); writing – review and editing (equal). **Gareth Evans:** Funding acquisition (equal); resources (equal); writing – review and editing (equal). **Peter Fasching:** Funding acquisition (equal); resources (equal); writing – review and editing (equal). **Tanja Fehm:** Funding acquisition (equal); resources (equal); writing – review and editing (equal). **Jonine Figueroa:** Funding acquisition (equal); resources (equal); writing – review and editing (equal). **Henrik Flyger:** Funding acquisition (equal); resources (equal); writing – review and editing (equal). **Marike Gabrielson:** Funding acquisition (equal); resources (equal); writing – review and editing (equal). **Manuela Gago‐Dominguez:** Funding acquisition (equal); resources (equal); writing – review and editing (equal). **Montserrat Garcia‐Closas:** Funding acquisition (equal); resources (equal); writing – review and editing (equal). **Jose A. Garcia‐Saenz:** Funding acquisition (equal); resources (equal); writing – review and editing (equal). **Jeanine Genkinger:** Funding acquisition (equal); resources (equal); writing – review and editing (equal). **Felix Grassmann:** Funding acquisition (equal); resources (equal); writing – review and editing (equal). **Melanie Gündert:** Funding acquisition (equal); resources (equal); writing – review and editing (equal). **Eric Hahnen:** Funding acquisition (equal); resources (equal); writing – review and editing (equal). **Christopher A. Haiman:** Funding acquisition (equal); resources (equal); writing – review and editing (equal). **Ute Hamann:** Funding acquisition (equal); resources (equal); writing – review and editing (equal). **Patricia A. Harrington:** Funding acquisition (equal); resources (equal); writing – review and editing (equal). **Jaana M. Hartikainen:** Funding acquisition (equal); resources (equal); writing – review and editing (equal). **Reiner Hoppe:** Funding acquisition (equal); resources (equal); writing – review and editing (equal). **John L. Hopper:** Funding acquisition (equal); resources (equal); writing – review and editing (equal). **Richard Houlston:** Funding acquisition (equal); resources (equal); writing – review and editing (equal). **Anthony Howell:** Funding acquisition (equal); resources (equal); writing – review and editing (equal). **ABCTB Investigators:** Funding acquisition (equal); resources (equal); writing – review and editing (equal). **kConFab investigators:** Funding acquisition (equal); resources (equal); writing – review and editing (equal). **Anna Jakubowska:** Funding acquisition (equal); resources (equal); writing – review and editing (equal). **Wolfgang Janni:** Funding acquisition (equal); resources (equal); writing – review and editing (equal). **Helena Jernström:** Funding acquisition (equal); resources (equal); writing – review and editing (equal). **Esther John:** Funding acquisition (equal); resources (equal); writing – review and editing (equal). **Nicola Johnson:** Funding acquisition (equal); resources (equal); writing – review and editing (equal). **Michael E. Jones:** Funding acquisition (equal); resources (equal); writing – review and editing (equal). **Vessela Nedelcheva Kristensen:** Funding acquisition (equal); resources (equal); writing – review and editing (equal). **Allison Kurian:** Funding acquisition (equal); resources (equal); writing – review and editing (equal). **Diether Lambrechts:** Funding acquisition (equal); resources (equal); writing – review and editing (equal). **Loic Le Marchand:** Funding acquisition (equal); resources (equal); writing – review and editing (equal). **Annika Lindblom:** Funding acquisition (equal); resources (equal); writing – review and editing (equal). **Jan Lubinski:** Funding acquisition (equal); resources (equal); writing – review and editing (equal). **Michael Patrick Lux:** Funding acquisition (equal); resources (equal); writing – review and editing (equal). **Arto Mannermaa:** Funding acquisition (equal); resources (equal); writing – review and editing (equal). **Dimitrios A Mavroudis:** Funding acquisition (equal); resources (equal); writing – review and editing (equal). **Anna Marie Mulligan:** Funding acquisition (equal); resources (equal); writing – review and editing (equal). **Taru Muranen:** Funding acquisition (equal); resources (equal); writing – review and editing (equal). **Heli Nevanlinna:** Funding acquisition (equal); resources (equal); writing – review and editing (equal). **Ines Nevelsteen:** Funding acquisition (equal); resources (equal); writing – review and editing (equal). **Patrick Neven:** Funding acquisition (equal); resources (equal); writing – review and editing (equal). **William G. Newman:** Funding acquisition (equal); resources (equal); writing – review and editing (equal). **Nadia Obi:** Funding acquisition (equal); resources (equal); writing – review and editing (equal). **Kenneth Offit:** Funding acquisition (equal); resources (equal); writing – review and editing (equal). **Andrew Olshan:** Funding acquisition (equal); resources (equal); writing – review and editing (equal). **Tjoung‐Won Park‐Simon:** Funding acquisition (equal); resources (equal); writing – review and editing (equal). **Alpa V Patel:** Funding acquisition (equal); resources (equal); writing – review and editing (equal). **Paolo Peterlongo:** Funding acquisition (equal); resources (equal); writing – review and editing (equal). **Kelly‐Anne Phillips:** Funding acquisition (equal); resources (equal); writing – review and editing (equal). **Dijana Plaseska‐Karanfilska:** Funding acquisition (equal); resources (equal); writing – review and editing (equal). **Eric C. Polley:** Funding acquisition (equal); resources (equal); writing – review and editing (equal). **Nadege Presneau:** Funding acquisition (equal); resources (equal); writing – review and editing (equal). **Katri Pylkäs:** Funding acquisition (equal); resources (equal); writing – review and editing (equal). **Brigitte Rack:** Funding acquisition (equal); resources (equal); writing – review and editing (equal). **Paolo Radice:** Funding acquisition (equal); resources (equal); writing – review and editing (equal). **Muhammad Usman Rashid:** Funding acquisition (equal); resources (equal); writing – review and editing (equal). **Valerie Rhenius:** Funding acquisition (equal); resources (equal); writing – review and editing (equal). **Mark Robson:** Funding acquisition (equal); resources (equal); writing – review and editing (equal). **ATOCHA ROMERO:** Funding acquisition (equal); resources (equal); writing – review and editing (equal). **Emmanouil Saloustros:** Funding acquisition (equal); resources (equal); writing – review and editing (equal). **Elinore Sayer:** Funding acquisition (equal); resources (equal); writing – review and editing (equal). **Rita K. Schmutzler:** Funding acquisition (equal); resources (equal); writing – review and editing (equal). **Sabine Schuetze:** Funding acquisition (equal); resources (equal); writing – review and editing (equal). **Christopher Scott:** Funding acquisition (equal); resources (equal); writing – review and editing (equal). **Mitul Shah:** Funding acquisition (equal); resources (equal); writing – review and editing (equal). **Snezhana Smichkoska:** Funding acquisition (equal); resources (equal); writing – review and editing (equal). **Melissa Southey:** Funding acquisition (equal); resources (equal); writing – review and editing (equal). **William J. Tapper:** Funding acquisition (equal); resources (equal); writing – review and editing (equal). **Lauren Teras:** Funding acquisition (equal); resources (equal); writing – review and editing (equal). **Rob Tollenaar:** Funding acquisition (equal); resources (equal); writing – review and editing (equal). **Katarzyna Tomczyk:** Funding acquisition (equal); resources (equal); writing – review and editing (equal). **Ian Tomlinson:** Funding acquisition (equal); resources (equal); writing – review and editing (equal). **Melissa Troester:** Funding acquisition (equal); resources (equal); writing – review and editing (equal). **Celine Vachon:** Funding acquisition (equal); resources (equal); writing – review and editing (equal). **Elke M. van Veen:** Funding acquisition (equal); resources (equal); writing – review and editing (equal). **Qin Wang:** Data curation (equal); funding acquisition (equal); resources (equal); writing – review and editing (equal). **Camilla Wendt:** Funding acquisition (equal); resources (equal); writing – review and editing (equal). **Hans Wildiers:** Funding acquisition (equal); resources (equal); writing – review and editing (equal). **Robert Winqvist:** Funding acquisition (equal); resources (equal); writing – review and editing (equal). **Argyrios Ziogas:** Funding acquisition (equal); resources (equal); writing – review and editing (equal). **Per Hall:** Funding acquisition (equal); resources (equal); writing – review and editing (equal). **Paul Pharaoh:** Funding acquisition (equal); resources (equal); writing – review and editing (equal). **Muriel A. Adank:** Funding acquisition (equal); resources (equal); writing – review and editing (equal). **Antoinette Hollestelle:** Funding acquisition (equal); resources (equal); writing – review and editing (equal). **Marjanka K. Schmidt:** Conceptualization (equal); data curation (equal); formal analysis (equal); funding acquisition (equal); investigation (equal); methodology (equal); software (equal); supervision (equal); writing – original draft (equal); writing – review and editing (equal). **Maartje Hooning:** Conceptualization (equal); data curation (equal); formal analysis (equal); funding acquisition (equal); investigation (equal); methodology (equal); software (equal); supervision (equal); writing – original draft (equal); writing – review and editing (equal).

## CONFLICT OF INTEREST STATEMENT

Matthias W. Beckmann and Peter A. Fasching conduct research funded by Amgen, Novartis and Pfizer (not related to this study). Peter A. Fasching received Honoraria from Roche, Novartis and Pfizer (not related to this study). Allison W. Kurian's institution received a research funding from Myriad genetics for an unrelated project (not related to this study). Emmanouil Saloustros reported the following: honoraria from Amgen Hellas, Pfizer Hellas, and IPSEN (not related to this study) and support from Merck Greece and Pfizer Hellas for attending meetings (not related to this study); he participated in advisory boards in Greece (MSD Greece, AstraZeneca Greece, Gilead Sciences Hellas, Pfizer Hellas, Genesis Pharma), not related to this study; he is a PI in sponsored clinical trials: MSD Greece (not related to this study). The other authors declare no conflict of interest.

## FUNDING INFORMATION

BCAC is funded by the European Union's Horizon 2020 Research and Innovation Programme (Grant Nos. 634935 and 633784 for BRIDGES and B‐CAST respectively), and the PERSPECTIVE I&I project, funded by the Government of Canada through Genome Canada and the Canadian Institutes of Health Research, the Ministère de l’Économie et de l'Innovation du Québec through Genome Québec, the Quebec Breast Cancer Foundation. The EU Horizon 2020 Research and Innovation Programme funding source had no role in study design, data collection, data analysis, data interpretation or writing of the report. Additional funding for BCAC is provided via the Confluence project which is funded with intramural funds from the National Cancer Institute Intramural Research Program, National Institutes of Health. Genotyping of the OncoArray was funded by the NIH Grant U19 CA148065, and Cancer Research UK Grant C1287/A16563 and the PERSPECTIVE project supported by the Government of Canada through Genome Canada and the Canadian Institutes of Health Research (Grant GPH‐129344) and, the Ministère de l'Économie, Science et Innovation du Québec through Genome Québec and the PSRSIIRI‐701 grant, and the Quebec Breast Cancer Foundation. Funding for iCOGS came from: the European Community's Seventh Framework Programme under Grant Agreement Number 223175 (HEALTH‐F2‐2009‐223175) (COGS), Cancer Research UK (C1287/A10118, C1287/A10710, C12292/A11174, C1281/A12014, C5047/A8384, C5047/A15007, C5047/A10692, C8197/A16565), the National Institutes of Health (CA128978) and Post‐Cancer GWAS initiative (1U19 CA148537, 1U19 CA148065 and 1U19 CA148112—the GAME‐ON initiative), the US Department of Defence (W81XWH‐10‐1‐0341), the Canadian Institutes of Health Research (CIHR) for the CIHR Team in Familial Risks of Breast Cancer, and Komen Foundation for the Cure, the Breast Cancer Research Foundation, and the Ovarian Cancer Research Fund. The BRIDGES panel sequencing was supported by the European Union Horizon 2020 research and innovation program BRIDGES (Grant No. 634935) and the Wellcome Trust (v203477/Z/16/Z). The Australian Breast Cancer Family Study (ABCFS) was supported by Grant UM1 CA164920 from the National Cancer Institute (USA). The content of this manuscript does not necessarily reflect the views or policies of the National Cancer Institute or any of the collaborating centers in the Breast Cancer Family Registry (BCFR), nor does mention of trade names, commercial products, or organizations imply endorsement by the USA Government or the BCFR. The ABCFS was also supported by the National Health and Medical Research Council of Australia, the New South Wales Cancer Council, the Victorian Health Promotion Foundation (Australia) and the Victorian Breast Cancer Research Consortium. J.L.H. is a National Health and Medical Research Council (NHMRC) Senior Principal Research Fellow. M.C.S. is a NHMRC Senior Research Fellow. The ABCS study was supported by the Dutch Cancer Society [Grant Nos. NKI 2007‐3839; 2009 4363]. The Australian Breast Cancer Tissue Bank (ABCTB) was supported by the National Health and Medical Research Council of Australia, The Cancer Institute NSW and the National Breast Cancer Foundation. The AHS study is supported by the intramural research program of the National Institutes of Health, the National Cancer Institute (Grant No. Z01‐CP010119), and the National Institute of Environmental Health Sciences (Grant No. Z01‐ES049030). The work of the BBCC was partly funded by ELAN‐Fond of the University Hospital of Erlangen. The BBCS is funded by Cancer Research UK and Breast Cancer Now and acknowledges NHS funding to the NIHR Biomedical Research Centre, and the National Cancer Research Network (NCRN). The BCEES was funded by the National Health and Medical Research Council, Australia and the Cancer Council Western Australia and acknowledges funding from the National Breast Cancer Foundation (JS). For the BCFR‐NY, BCFR‐PA, BCFR‐UT this work was supported by Grant UM1 CA164920 from the National Cancer Institute. The content of this manuscript does not necessarily reflect the views or policies of the National Cancer Institute or any of the collaborating centers in the Breast Cancer Family Registry (BCFR), nor does mention of trade names, commercial products, or organizations imply endorsement by the US Government or the BCFR. The BCINIS study is supported in part by the Breast Cancer Research Foundation (BCRF). For BIGGS, ES is supported by NIHR Comprehensive Biomedical Research Centre, Guy's & St. Thomas' NHS Foundation Trust in partnership with King's College London, United Kingdom. IT is supported by the Oxford Biomedical Research Centre. The BREast Oncology GAlician Network (BREOGAN) is funded by Acción Estratégica de Salud del Instituto de Salud Carlos III FIS PI12/02125/Cofinanciado and FEDER PI17/00918/Cofinanciado FEDER; Acción Estratégica de Salud del Instituto de Salud Carlos III FIS Intrasalud (PI13/01136); Programa Grupos Emergentes, Cancer Genetics Unit, Instituto de Investigacion Biomedica Galicia Sur. Xerencia de Xestion Integrada de Vigo‐SERGAS, Instituto de Salud Carlos III, Spain; Grant 10CSA012E, Consellería de Industria Programa Sectorial de Investigación Aplicada, PEME I + D e I + D Suma del Plan Gallego de Investigación, Desarrollo e Innovación Tecnológica de la Consellería de Industria de la Xunta de Galicia, Spain; Grant EC11‐192. Fomento de la Investigación Clínica Independiente, Ministerio de Sanidad, Servicios Sociales e Igualdad, Spain; and Grant FEDER‐Innterconecta. Ministerio de Economia y Competitividad, Xunta de Galicia, Spain. The BSUCH study was supported by the Dietmar‐Hopp Foundation, the Helmholtz Society and the German Cancer Research Center (DKFZ). CCGP is supported by funding from the University of Crete. The CECILE study was supported by Fondation de France, Institut National du Cancer (INCa), Ligue Nationale contre le Cancer, Agence Nationale de Sécurité Sanitaire, de l'Alimentation, de l'Environnement et du Travail (ANSES), Agence Nationale de la Recherche (ANR). The CGPS was supported by the Chief Physician Johan Boserup and Lise Boserup Fund, the Danish Medical Research Council, and Herlev and Gentofte Hospital. The American Cancer Society funds the creation, maintenance, and updating of the CPS‐II cohort. The California Teachers Study (CTS) and the research reported in this publication were supported by the National Cancer Institute of the National Institutes of Health under award number U01‐CA199277; P30‐CA033572; P30‐CA023100; UM1‐CA164917; and R01‐CA077398. The content is solely the responsibility of the authors and does not necessarily represent the official views of the National Cancer Institute or the National Institutes of Health. The collection of cancer incidence data used in the California Teachers Study was supported by the California Department of Public Health pursuant to California Health and Safety Code Section 103885; Centers for Disease Control and Prevention's National Program of Cancer Registries, under cooperative agreement 5NU58DP006344; the National Cancer Institute's Surveillance, Epidemiology and End Results Program under contract HHSN261201800032I awarded to the University of California, San Francisco, contract HHSN261201800015I awarded to the University of Southern California, and contract HHSN261201800009I awarded to the Public Health Institute. The opinions, findings, and conclusions expressed herein are those of the author(s) and do not necessarily reflect the official views of the State of California, Department of Public Health, the National Cancer Institute, the National Institutes of Health, the Centers for Disease Control and Prevention or their Contractors and Subcontractors, or the Regents of the University of California, or any of its programs. The University of Westminster curates the DietCompLyf database funded by Against Breast Cancer Registered Charity No. 1121258 and the NCRN. The coordination of EPIC is financially supported by the European Commission (DG‐SANCO) and the International Agency for Research on Cancer. The national cohorts are supported by: Ligue Contre le Cancer, Institut Gustave Roussy, Mutuelle Générale de l’Education Nationale, Institut National de la Santé et de la Recherche Médicale (INSERM) (France); German Cancer Aid, German Cancer Research Center (DKFZ), Federal Ministry of Education and Research (BMBF) (Germany); the Hellenic Health Foundation, the Stavros Niarchos Foundation (Greece); Associazione Italiana per la Ricerca sul Cancro‐AIRC‐Italy and National Research Council (Italy); Dutch Ministry of Public Health, Welfare and Sports (VWS), Netherlands Cancer Registry (NKR), LK Research Funds, Dutch Prevention Funds, Dutch ZON (Zorg Onderzoek Nederland), World Cancer Research Fund (WCRF), Statistics Netherlands (the Netherlands); Health Research Fund (FIS), PI13/00061 to Granada, PI13/01162 to EPIC‐Murcia, Regional Governments of Andalucía, Asturias, Basque Country, Murcia and Navarra, ISCIII RETIC (RD06/0020) (Spain); Cancer Research UK (14136 to EPIC‐Norfolk; C570/A16491 and C8221/A19170 to EPIC‐Oxford), Medical Research Council (1000143 to EPIC‐Norfolk, MR/M012190/1 to EPIC‐Oxford) (United Kingdom). The ESTHER study was supported by a grant from the Baden Württemberg Ministry of Science, Research and Arts.Additional cases were recruited in the context of the VERDI study, which was supported by a grant from the German Cancer Aid (Deutsche Krebshilfe). FHRISK and PROCAS are funded from NIHR Grant PGfAR 0707‐10031. DGE, AH and WGN are supported by the NIHR Manchester Biomedical Research Centre (IS‐BRC‐1215‐20007). The GC‐HBOC (German Consortium of Hereditary Breast and Ovarian Cancer) is supported by the German Cancer Aid (Grant Nos. 110837 and 70114178, coordinator: Rita K. Schmutzler, Cologne) and the Federal Ministry of Education and Research, Germany (Grant No. 01GY1901). This work was also funded by the European Regional Development Fund and Free State of Saxony, Germany (LIFE—Leipzig Research Centre for Civilization Diseases, Project numbers 713‐241202, 713‐241202, 14505/2470, 14575/2470). The GENICA was funded by the Federal Ministry of Education and Research (BMBF) Germany Grants 01KW9975/5, 01KW9976/8, 01KW9977/0 and 01KW0114, the Robert Bosch Foundation, Stuttgart, Deutsches Krebsforschungszentrum (DKFZ), Heidelberg, the Institute for Prevention and Occupational Medicine of the German Social Accident Insurance, Institute of the Ruhr University Bochum (IPA), Bochum, as well as the Department of Internal Medicine, Johanniter GmbH Bonn, Johanniter Krankenhaus, Bonn, Germany. The GESBC was supported by the Deutsche Krebshilfe e. V. [70492] and the German Cancer Research Center (DKFZ). The HABCS study was supported by the Claudia von Schilling Foundation for Breast Cancer Research, by the Lower Saxonian Cancer Society, and by the Rudolf Bartling Foundation. The HEBCS was financially supported by the Helsinki University Hospital Research Fund, the Sigrid Juselius Foundation and the Cancer Foundation Finland. Financial support for KARBAC was provided through the regional agreement on medical training and clinical research (ALF) between Stockholm County Council and Karolinska Institutet, the Swedish Cancer Society, The Gustav V Jubilee foundation and Bert von Kantzows foundation. The KARMA study was supported by Märit and Hans Rausings Initiative Against Breast Cancer. The KBCP was financially supported by the special Government Funding (VTR) of Kuopio University Hospital grants, Cancer Fund of North Savo, the Finnish Cancer Organizations, and by the strategic funding of the University of Eastern Finland. kConFab and the kConFab Follow‐Up Study have been supported by grants from Cancer Australia (809195 and 1100868), the Australian National Breast Cancer Foundation (IF 17 kConFab), the National Health and Medical Research Council (454508, 288704, and 145684), the Queensland Cancer Fund, the Cancer Councils of New South Wales, Victoria, Tasmania, and South Australia, and the Cancer Foundation of Western Australia. K‐AP is a National Health and Medical Research Council Leadership Fellow (Australia) (1195294). Financial support for the AOCS was provided by the United States Army Medical Research and Materiel Command [DAMD17‐01‐1‐0729], Cancer Council Victoria, Queensland Cancer Fund, Cancer Council New South Wales, Cancer Council South Australia, The Cancer Foundation of Western Australia, Cancer Council Tasmania and the National Health and Medical Research Council of Australia (NHMRC; 400413, 400281, 199600). G.C.T. and P.W. are supported by the NHMRC. RB was a Cancer Institute NSW Clinical Research Fellow. LMBC is supported by the “Stichting tegen Kanker.” DL is supported by the FWO. The MABCS study is funded by the Research Centre for Genetic Engineering and Biotechnology “Georgi D. Efremov”, MASA. The MARIE study was supported by the Deutsche Krebshilfe e.V. [70‐2892‐BR I, 106332, 108253, 108419, 110826, 110828], the Hamburg Cancer Society, the German Cancer Research Center (DKFZ) and the Federal Ministry of Education and Research (BMBF) Germany [01KH0402]. MBCSG is supported by grants from the Italian Association for Cancer Research (AIRC). The MCBCS was supported by the NIH Grants R35CA253187, R01CA192393, R01CA116167, R01CA176785 a NIH Specialized Program of Research Excellence (SPORE) in Breast Cancer [P50CA116201], and the Breast Cancer Research Foundation. The Melbourne Collaborative Cohort Study (MCCS) cohort recruitment was funded by VicHealth and Cancer Council Victoria. The MCCS was further augmented by Australian National Health and Medical Research Council Grants 209057, 396414 and 1074383 and by infrastructure provided by Cancer Council Victoria. Cases and their vital status were ascertained through the Victorian Cancer Registry and the Australian Institute of Health and Welfare, including the National Death Index and the Australian Cancer Database. The MEC was supported by NIH Grants CA63464, CA54281, CA098758, CA132839 and CA164973. The MISS study was supported by funding from ERC‐2011‐294576 Advanced grant, Swedish Cancer Society CAN 2018/675, Swedish Research Council, Local hospital funds, Berta Kamprad Foundation FBKS 2021‐19, Gunnar Nilsson. The MMHS study was supported by NIH Grants CA97396, CA128931, CA116201, CA140286 and CA177150. MSKCC is supported by grants from the Breast Cancer Research Foundation and Robert and Kate Niehaus Clinical Cancer Genetics Initiative The NBCS has received funding from the K.G. Jebsen Centre for Breast Cancer Research; the Research Council of Norway Grant 193387/V50 (to A‐L Børresen‐Dale and V.N. Kristensen) and Grant 193387/H10 (to A‐L Børresen‐Dale and V.N. Kristensen), South Eastern Norway Health Authority (Grant 39346 to A‐L Børresen‐Dale) and the Norwegian Cancer Society (to A‐L Børresen‐Dale and V.N. Kristensen). The Northern California Breast Cancer Family Registry (NC‐BCFR) and Ontario Familial Breast Cancer Registry (OFBCR) were supported by Grant U01CA164920 from the USA National Cancer Institute of the National Institutes of Health. The content of this manuscript does not necessarily reflect the views or policies of the National Cancer Institute or any of the collaborating centers in the Breast Cancer Family Registry (BCFR), nor does mention of trade names, commercial products, or organizations imply endorsement by the USA Government or the BCFR. The Carolina Breast Cancer Study (NCBCS) was funded by Komen Foundation, the National Cancer Institute (P50 CA058223, U54 CA156733, U01 CA179715), and the North Carolina University Cancer Research Fund. The NHS was supported by NIH Grants P01 CA87969, UM1 CA186107, and U19 CA148065. The NHS2 was supported by NIH Grants UM1 CA176726 and U19 CA148065. The OBCS was supported by research grants from the Finnish Cancer Foundation, the Academy of Finland (Grant Nos. 250083, 122715 and Center of Excellence Grant No. 251314), the Finnish Cancer Foundation, the Sigrid Juselius Foundation, the University of Oulu, the University of Oulu Support Foundation and the special Governmental EVO funds for Oulu University Hospital‐based research activities. The ORIGO study was supported by the Dutch Cancer Society (RUL 1997‐1505) and the Biobanking and Biomolecular Resources Research Infrastructure (BBMRI‐NL CP16). The PBCS was funded by Intramural Research Funds of the National Cancer Institute, Department of Health and Human Services, USA. Genotyping for PLCO was supported by the Intramural Research Program of the National Institutes of Health, NCI, Division of Cancer Epidemiology and Genetics. The PLCO is supported by the Intramural Research Program of the Division of Cancer Epidemiology and Genetics and supported by contracts from the Division of Cancer Prevention, National Cancer Institute, National Institutes of Health. The POSH study is funded by Cancer Research UK (Grant Nos. C1275/A11699, C1275/C22524, C1275/A19187, C1275/A15956 and Breast Cancer Campaign 2010PR62, 2013PR044. The RBCS was funded by the Dutch Cancer Society (DDHK 2004–3124, DDHK 2009‐4318). The SASBAC study was supported by funding from the Agency for Science, Technology and Research of Singapore (A*STAR), the US National Institute of Health (NIH) and the Susan G. Komen Breast Cancer Foundation. The SBCS was supported by Sheffield Experimental Cancer Medicine Centre and Breast Cancer Now Tissue Bank. SEARCH is funded by Cancer Research UK [C490/A10124, C490/A16561] and supported by the UK National Institute for Health Research Biomedical Research Centre at the University of Cambridge. The University of Cambridge has received salary support for PDPP from the NHS in the East of England through the Clinical Academic Reserve. The SZBCS was supported by Grant PBZ_KBN_122/P05/2004 and the program of the Minister of Science and Higher Education under the name “Regional Initiative of Excellence” in 2019–2022 project number 002/RID/2018/19 amount of financing 12 000 000 PLN. UBCS was supported by funding from National Cancer Institute (NCI) Grant R01 CA163353 (to N.J. Camp) and the Women's Cancer Center at the Huntsman Cancer Institute (HCI). Data collection for UBCS was supported by the Utah Population Database (UPDB) and Utah Cancer Registry (UCR). Partial support for UPDB datasets was provided by the University of Utah HCI and the HCI Cancer Center Support Grant, P30 CA2014 from the National Cancer Institute. UCR is funded by the National Cancer Institute's SEER Program, Contract No. HHSN261201800016I, the US Centers for Disease Control and Prevention's National Program of Cancer Registries, Cooperative Agreement No. NU58DP006320, with additional support from the University of Utah and Huntsman Cancer Foundation. The UCIBCS component of this research was supported by the NIH [CA58860, CA92044] and the Lon V Smith Foundation [LVS39420]. The UKBGS is funded by Breast Cancer Now andthe Institute of Cancer Research (ICR), London. ICR acknowledges NHS funding to the NIHR Biomedical Research Centre. The US3SS study was supported by Massachusetts (K.M.E., R01CA47305), Wisconsin (P.A.N., R01 CA47147) and New Hampshire (L.T.‐E., R01CA69664) centers, and Intramural Research Funds of the National Cancer Institute, Department of Health and Human Services, USA. The USRT Study was funded by Intramural Research Funds of the National Cancer Institute, Department of Health and Human Services, USA.

## ETHICS STATEMENT

All individual studies were approved by the appropriate institutional review boards and/or medical ethical committees. Written informed consent was obtained from all study participants.

## Supporting information


Supplementary Methods.

Table S1.

Tables S2–S18.

## Data Availability

The datasets analyzed during the current study are not publicly available due to protection of participant privacy and confidentiality, and ownership of the contributing institutions, but may be made available in an anonymized form via the corresponding author on reasonable request and after approval of the involved institutions. To receive access to the data, a concept form must be submitted, which will then be reviewed by the BCAC Data Access Coordination Committee (DACC); see http://bcac.ccge.medschl.cam.ac.uk/bcacdata/.

## References

[1] Sung H , Ferlay J , Siegel RL , et al. Global cancer statistics 2020: GLOBOCAN estimates of incidence and mortality worldwide for 36 cancers in 185 countries. CA Cancer J Clin. 2021;71:209‐249. 33538338 10.3322/caac.21660

[2] CHEK2 Breast Cancer Case‐Control Consortium . CHEK2*1100delC and susceptibility to breast cancer: a collaborative analysis involving 10,860 breast cancer cases and 9,065 controls from 10 studies. Am J Hum Genet. 2004;74:1175‐1182. 15122511 10.1086/421251 PMC1182081

[3] Schmidt MK , Hogervorst F , van Hien R , et al. Age‐ and tumor subtype‐specific breast cancer risk estimates for CHEK2*1100delC carriers. J Clin Oncol. 2016;34:2750‐2760. 27269948 10.1200/JCO.2016.66.5844 PMC5019754

[4] Weischer M , Nordestgaard BG , Pharoah P , et al. CHEK2*1100delC heterozygosity in women with breast cancer associated with early death, breast cancer‐specific death, and increased risk of a second breast cancer. J Clin Oncol. 2012;30:4308‐4316. 23109706 10.1200/JCO.2012.42.7336 PMC3515767

[5] Thompson D , Seal S , Schutte M , et al. A multicenter study of cancer incidence in CHEK2 1100delC mutation carriers. Cancer Epidemiol Biomarkers Prev. 2006;15:2542‐2545. 17164383 10.1158/1055-9965.EPI-06-0687 PMC2714971

[6] de Bock GH , Schutte M , Krol‐Warmerdam EM , et al. Tumour characteristics and prognosis of breast cancer patients carrying the germline CHEK2*1100delC variant. J Med Genet. 2004;41:731‐735. 15466005 10.1136/jmg.2004.019737 PMC1735606

[7] Perou CM , Sorlie T , Eisen MB , et al. Molecular portraits of human breast tumours. Nature. 2000;406:747‐752. 10963602 10.1038/35021093

[8] Broeks A , de Witte L , Nooijen A , et al. Excess risk for contralateral breast cancer in CHEK2*1100delC germline mutation carriers. Breast Cancer Res Treat. 2004;83:91‐93. 14997059 10.1023/B:BREA.0000010697.49896.03

[9] Greville‐Heygate SL , Maishman T , Tapper WJ , et al. Pathogenic variants in CHEK2 are associated with an adverse prognosis in symptomatic early‐onset breast cancer. JCO Precis Oncol. 2020;4:485. 10.1200/PO.19.00178 PMC7446368 32923877

[10] Akdeniz D , Schmidt MK , Seynaeve CM , et al. Risk factors for metachronous contralateral breast cancer: a systematic review and meta‐analysis. Breast. 2019;44:1‐14. 30580169 10.1016/j.breast.2018.11.005

[11] Thompson LH . Recognition, signaling, and repair of DNA double‐strand breaks produced by ionizing radiation in mammalian cells: the molecular choreography. Mutat Res. 2012;751:158‐246. 22743550 10.1016/j.mrrev.2012.06.002

[12] Bartek J , Lukas J . Chk1 and Chk2 kinases in checkpoint control and cancer. Cancer Cell. 2003;3:421‐429. 12781359 10.1016/s1535-6108(03)00110-7

[13] Broeks A , Braaf LM , Huseinovic A , et al. Identification of women with an increased risk of developing radiation‐induced breast cancer: a case only study. Breast Cancer Res. 2007;9:R26. 17428320 10.1186/bcr1668 PMC1868917

[14] Kramer I , Schaapveld M , Oldenburg HSA , et al. The influence of adjuvant systemic regimens on contralateral breast cancer risk and receptor subtype. J Natl Cancer Inst. 2019;111:709‐718. 30698719 10.1093/jnci/djz010

[15] Kriege M , Hollestelle A , Jager A , et al. Survival and contralateral breast cancer in CHEK2 1100delC breast cancer patients: impact of adjuvant chemotherapy. Br J Cancer. 2014;111:1004‐1013. 24918820 10.1038/bjc.2014.306 PMC4150261

[16] Dorling L , Carvalho S , Allen J , et al. Breast cancer risk genes—association analysis in more than 113,000 women. N Engl J Med. 2021;384:428‐439. 33471991 10.1056/NEJMoa1913948 PMC7611105

[17] Adank MA , Jonker MA , Kluijt I , et al. CHEK2*1100delC homozygosity is associated with a high breast cancer risk in women. J Med Genet. 2011;48:860‐863. 22058428 10.1136/jmedgenet-2011-100380

[18] Amos CI , Dennis J , Wang Z , et al. The OncoArray Consortium: a network for understanding the genetic architecture of common cancers. Cancer Epidemiol Biomarkers Prev. 2017;26:126‐135. 27697780 10.1158/1055-9965.EPI-16-0106 PMC5224974

[19] Bahcall O . COGS project and design of the iCOGS array. Nat Genet. 2013;45(4):343. 23535721 10.1038/ng.2592

[20] Early Breast Cancer Trialists' Collaborative Group . Effects of chemotherapy and hormonal therapy for early breast cancer on recurrence and 15‐year survival: an overview of the randomised trials. Lancet. 2005;365:1687‐1717. 15894097 10.1016/S0140-6736(05)66544-0

[21] Mellemkjaer L , Dahl C , Olsen JH , et al. Risk for contralateral breast cancer among carriers of the CHEK2*1100delC mutation in the WECARE study. Br J Cancer. 2008;98:728‐733. 18253122 10.1038/sj.bjc.6604228 PMC2259175

[22] Kriege M , Jager A , Hollestelle A , et al. Sensitivity to systemic therapy for metastatic breast cancer in CHEK2 1100delC mutation carriers. J Cancer Res Clin Oncol. 2015;141:1879‐1887. 25958056 10.1007/s00432-015-1981-7 PMC4543421

[23] Hooning MJ , Aleman BM , Hauptmann M , et al. Roles of radiotherapy and chemotherapy in the development of contralateral breast cancer. J Clin Oncol. 2008;26:5561‐5568. 18854572 10.1200/JCO.2007.16.0192

[24] Stovall M , Smith SA , Langholz BM , et al. Dose to the contralateral breast from radiotherapy and risk of second primary breast cancer in the WECARE study. Int J Radiat Oncol Biol Phys. 2008;72:1021‐1030. 18556141 10.1016/j.ijrobp.2008.02.040 PMC3782859

[25] Boice JD Jr , Harvey EB , Blettner M , Stovall M , Flannery JT . Cancer in the contralateral breast after radiotherapy for breast cancer. N Engl J Med. 1992;326:781‐785. 1538720 10.1056/NEJM199203193261201

[26] van Buuren S . Flexible Imputation of Missing Dataed. CRC Press; 2012.

[27] Kyriacou DN , Lewis RJ . Confounding by indication in clinical research. JAMA. 2016;316:1818‐1819. 27802529 10.1001/jama.2016.16435

