# Supplementary material for: Association of the CHEK2 c.1100delC variant, radiotherapy, and systemic treatment with contralateral breast cancer risk and breast cancer‐specific survival
Source: Cancer Med. 2023 Jul 3;12(15):16142–62. doi: 10.1002/cam4.6272 (PMC10469654; doi:10.1002/cam4.6272)
Supplement: Supplementary file 1 — Supplementary Methods. [file CAM4-12-16142-s001.docx]

**Supplementary methods**

*CHEK2* c.1100delC genotyping

The *CHEK2* c.1100delC genotypes were obtained from five different sources: BRIDGES sequencing data [1], Taqman, iPLEX [2, 3], and imputed dosages using the OncoArray [4] and iCOGS [5] SNP-arrays using the 1000 Genomes Project Phase 3 (October 2014) release as reference panel [6]. Given that some study subjects had information from different genotyping platforms, we constructed a combined genotype variable according to the following hierarchy: BRIDGES genotype, followed by Taqman and iPLEX; imputed OncoArray dosage if none of the previous was available; imputed iCOGS dosage if none of the previous was available. We only considered heterozygous carriers (*CHEK2* c.1100delC genotype = 1). The few homozygous carriers identified were excluded (Figure 1). For imputed dosages, we considered individuals as carriers if the corresponding dosage was ≥1 and <2.

Multiple imputation of missing data

Multiple imputation was performed to address the presence of missing data in several clinical and pathological variables included as covariates in the multivariable Cox regression models. The R package MICE (version 3.13.0) was used to impute 10 datasets through 30 iterations of the multivariate imputation by chained equations (MICE) algorithm. Imputation was performed on a total of 100,973 breast cancer patients (Supplementary Table S1) after initial exclusion criteria as specified in Figure 1.

The list of imputed variables, corresponding percentage of missing values, imputation methods, and information about pre-processing of the data can be found in Supplementary Table S2. Variables included in the imputation process were imputed according to the number of missing values, from the least to the most missing.

For each imputed variable, predictors in the corresponding imputation model were selected among all the variables included in the imputation process based on the correlation coefficient with the variable to impute (≥0.125) and the proportion of observed values among the cases with missing data on the variable to impute (≥0.200). In particular, neo-adjuvant chemotherapy status (yes vs no) was added as predictor in the imputation models of neo-adjuvant anthracyclines status, neo-adjuvant taxanes status and neo-adjuvant CMF-like chemotherapy status. Year of diagnosis was included as predictor in the imputation model of surgery. The variable “study” was included in all imputation models, in order to preserve the heterogeneity among studies as much as possible, and because in case of systematic missing values (variables not measured/reported by entire studies) it is an informative predictor, which needs to be included to fulfill the missing at random assumption [7].

The Nelson-Aalen estimator of the baseline cumulative hazard and the event indicator of both overall survival and breast cancer-specific survival were included in all imputation models to improve imputation [8], as well as the time to contralateral breast cancer (CBC) and the corresponding event indicator. Both CBC status and time to CBC had missing values and were therefore also imputed; however the imputed values for these two variables were not used in the analyses.

Estimates from the analyses across different imputed datasets were combined via Rubin’s rule [9, 10].

Multi-state model

To get further insight into the relation between CHEK2 c.1100delC status, treatment given for the first BC, CBC risk and death, we used a multi-state model in the framework of the Cox model, with diagnosis of the first BC as initial state, diagnosis of CBC as intermediate (transient) state, and death due to BC, death due to other causes, and death due to unknown causes as absorbing states (Figure 2). For all direct transitions from the initial state, time at risk started either three months after first BC diagnosis or at study entry if study entry was more than three months after first BC diagnosis. The time-to-state started at the time the patient entered the initial state and moved forward through the occurrence of intermediate events, if any (“clock forward” approach) [11]. As for the CBC risk analyses, we assumed that patients with unknown CBC status did not develop a CBC during follow-up, and that for CBC cases with unknown time from first BC to CBC diagnosis, CBC occurrence was at last available follow-up. The baseline hazard was allowed to vary across country and transition. Next to the CHEK2 c.1100delC status, radiation and (type of) systemic treatment given for the first BC, also age at diagnosis, tumor size, nodal status, grade, and ER status for the first BC were included in the multi-state model as covariates. In order to investigate the effect of each variable on the different endpoints, the HR estimates were allowed to vary across transition.

1. Dorling, L., et al., *Breast Cancer Risk Genes - Association Analysis in More than 113,000 Women.* N Engl J Med, 2021. **384**(5): p. 428-439.

2. Weischer, M., et al., *CHEK2*1100delC heterozygosity in women with breast cancer associated with early death, breast cancer-specific death, and increased risk of a second breast cancer.* J Clin Oncol, 2012. **30**(35): p. 4308-16.

3. Schmidt, M.K., et al., *Age- and Tumor Subtype-Specific Breast Cancer Risk Estimates for CHEK2*1100delC Carriers.* J Clin Oncol, 2016. **34**(23): p. 2750-60.

4. Amos, C.I., et al., *The OncoArray Consortium: A Network for Understanding the Genetic Architecture of Common Cancers.* Cancer Epidemiol Biomarkers Prev, 2017. **26**(1): p. 126-135.

5. Bahcall, O., *COGS project and design of the iCOGS array.* Nature Genetics, 2013.

6. Auton, A., et al., *A global reference for human genetic variation.* Nature, 2015. **526**(7571): p. 68-74.

7. Buuren, S.v., *Flexible imputation of missing data*. Second edition ed. 2018: Chapman & Hall/CRC.

8. White, I.R. and P. Royston, *Imputing missing covariate values for the Cox model.* Stat Med, 2009. **28**(15): p. 1982-98.

9. Barnard, J. and D.B. Rubin, *Small-Sample Degrees of Freedom with Multiple Imputation.* Biometrika, 1999. **86**(4): p. 948-955.

10. Rubin, D.B., *Multiple Imputation for Nonresponse in Surveys*. Wiley Series in Probability and Statistics. 1987, New York: Wiley.

11. Putter, H., M. Fiocco, and R.B. Geskus, *Tutorial in biostatistics: competing risks and multi-state models.* Statistics in Medicine, 2007. **26**(11): p. 2389-2430.
