# Supplementary material for: Association of the CHEK2 c.1100delC variant, radiotherapy, and systemic treatment with contralateral breast cancer risk and breast cancer‐specific survival
Source: Cancer Med. 2023 Jul 3;12(15):16142–62. doi: 10.1002/cam4.6272 (PMC10469654; doi:10.1002/cam4.6272)
Supplement: Supplementary file 2 — Table S1. [file CAM4-12-16142-s002.docx]

**Table S1.** Overview of the Breast Cancer Association Consortium (BCAC) studies included in the analyses.

| **Study (abbreviation)** | **Country** | **Study design** | **Case definition** | **Design category** | **How follow-up (including vital status) information was obtained** | **Selected familial cases** | **Total Cases included in the main analyses** | **Total cases included for multiple imputation** | **Age (cases) range*** |
| --- | --- | --- | --- | --- | --- | --- | --- | --- | --- |
| Australian Breast Cancer Family Study  (ABCFS) | Australia | Population-based case-control study | All cases diagnosed < age 40 plus a random sample of those diagnosed ages 40-59 from cancer registries in Victoria and New South Wales, plus a limited number diagnosed aged 60-69; cases living in Melbourne recruited from 1992-99 and in Sydney from 1993-98. | Population-based case-control | Systematic follow-ups by mail and telephone | No | 1287 | 1307 | 23 - 84 |
| Amsterdam Breast Cancer Study  (ABCS) | Netherlands | Hospital-based consecutive cases; population-based controls (for iCOGS/OncoArray from blood bank). | Pre-iCOGS:  All breast cancer patients (with operable, invasive mammacarcinoma) aged <50 years and diagnosed from 1970-1994 in four Dutch hospitals.  iCOGS/OncoArray:  Breast cancer patients diagnosed before age 50 in 1995-2011 at the Netherlands Cancer Insitute - Antoni van Leeuwenhoek hospital (NKI-AVL). | Mixed | Hospital medical registry and linkage with municipality registry | No | 2155 | 2212 | 18 - 77 |
| Amsterdam Breast Cancer Study – Familial  (ABCS-F) | Netherlands | Clinical Genetic Center-based cases | Pre-iCOGS:  Only in BCAC PhaseI/II familial non-BRCA1/2 cases <50 from the Clinical Genetic Centre of the Netherlands Cancer Institute were included.  iCOGS/OncoArray: All non-BRCA1/2 breast cancer cases from the family cancer clinic of the NKI-AVL tested in the period 1995-2009; all ages and diagnosed with breast cancer in 1965-2012. | Case-series | Hospital medical registry and linkage with municipality registry | Yes | 434 | 489 | 23 - 79 |
| Australian Breast Cancer Tissue Bank  (ABCTB) | Australia | Hospital-based multi site newly diagnosed breast cancer case | Newly diagnosed unselected cases from 32 hospitals in New South Wales from 2006 | Mixed | Multiple (medical records at surgeron's rooms, medical records and databases at hospital clinics and GP Clincs) | No | 700 | 715 | 26 - 97 |
| Agricultural Health Study  (AHS) | USA | Prospective cohort study: nested case-control | This is a nested case-control study conducted within a cohort of pesticide (57,310) applicators and their spouses (n=32, 345) who enrolled during 1993-1997 in Iowa and North Carolina. Cases are white females with incident breast cancer diagnosed between 1993 and 2012 in North Carolina and 2013 in Iowa with no previous history of any cancer. | Nested case-control | Vital staus was obtained through linkage with state mortality registries and the National Death Index. Follow-up demogrpahic and risk factor information was collected via questionnair over the course of study follow-up. | No | 0 | 512 | 31 - 89 |
| Bavarian Breast Cancer Cases and Controls  (BBCC) | Germany | Hospital-based cases; population based controls | Consecutive, unselected cases with invasive breast cancer recruited at the University Breast Centre, Franconia in Northern Bavaria during 1999-2013. | Mixed | Cancer registry and Medical records. | No | 1414 | 1549 | 19 - 95 |
| Breast Cancer Employment and Environment Study  (BCEES) | Australia | Population-based case-control study | First incident invasive breast cancer diagnosed between May 2009 and January 2011, residing in Western Australia and reported to the state wide mandatory Cancer Registry. | Population-based case-control | No follow-up has been completed | No | 0 | 783 | 27 - 80 |
| New York Breast Cancer Family Registry  (BCFR-NY) | USA | Clinic-based recruitment of families; family-based cohort | Recruitment took place from Jan 1996 to Dec 2012. Eligibility was based on one or more of the following criteria: two or more relatives with a personal history of breast or ovarian cancer; a woman diagnosed with breast or ovarian cancer at a young age; a woman with a history of both breast and ovarian cancer; an affected male; or known BRCA1 or BRCA2 mutation carriers. | Mixed | Systematic follow-up every five years for questionnaire data and annual update of cancer history and vital records by at least one family member | Yes | 304 | 337 | 23 - 78 |
| Philadelphia Breast Cancer Family Registry  (BCFR-PA) | USA | Clinic-based recruitment of families; family-based cohort | Recruitment took place from 1996 to 2011. Eligibility was based on one or more of the following criteria: 2 or more relatives with a personal history of breast or ovarian cancer; a woman diagnosed with breast or ovarian cancer at a young age; a woman with a history of both breast and ovarian cancer; an affected male; or known BRCA1 or BRCA1 mutation carriers. | Case-series | Self-report on questionnaires or clinical database | Yes | 43 | 46 | 24 - 56 |
| Utah Breast Cancer Family Registry  (BCFR-UTAH) | USA | Clinic-based recruitment of non-BRCA1/2 familial breast cancer cases;unaffected BRCA1/2 carriers as controls | Index cases from families tested negative for BRCA1/2 mutations. Recruited in Utah during 1995-2008. | Mixed | Follow-up questionnaire | Yes | 14 | 90 | 28 - 74 |
| Breast Cancer in Northern Israel Study  (BCINIS) | Israel | Population-based case-control study | All consecutive cases of invasive BC and DCIS diagnosed in a geographically defined area of Northern Israel since 1990. On-going. Includes a founder population with mutations responsible for 10% of all cases. | Population-based case-control | Oncology files, medical records, population database | No | 0 | 1048 | 24 - 97 |
| Breast Cancer in Galway Genetic Study  (BIGGS) | Ireland | Hospital-based cases; population based controls | Unselected cases recruited from West of Ireland since 2001. Cases were recruited from University College Hospital Galway and surrounding hospitals | Mixed | Local clinical database | No | 512 | 532 | 24 - 89 |
| Breast Oncology Galicia Network  (BREOGAN) | Spain | Population-based case-control | A population-based study conducted since 1997 in two cities in Galicia, Spain (Vigo and Santiago) covering approximately 700,000 inhabitants. The study currently includes over 1600 incident breast cancer cases diagnosed from 1997-2014 in two Galician hospitals with blood, tumor tissue and risk factor questionnaire. | Mixed | From the computerised electronic individual medical history program unique for each individual in the state: Each individual is identified by his/her social security number which is unique to each person during his/her lifetime, his/her first name and his/her two last names, his/her national id number which is unique to each individual during his/her lifetime, date of bith, etc: All information is 100% computerised under the Ianus program. This program includes information from all services and departments in all state hospitals, continuing update from all hospitals and family clinics as well as electronic prescriptions and medical imaging. | No | 1501 | 1517 | 25 - 94 |
| Breast Cancer Study of the University of Heidelberg  (BSUCH) | Germany | Hospital-based cases;healthy blood donator controls | Cases diagnosed with breast cancer/breast cancer metastasis in 2008-2011 at the University Women`s Clinic Heidelberg. | Mixed | Individual clinical investigation of patients, medical records | No | 878 | 911 | 25 - 88 |
| Crete Cancer Genetics Program  (CCGP) | Greece | Hospital-based case-control study | Incident breast cancer cases treated between 2004 and 2013 at the University Hospital of Heraklion on Crete; all enrolled within 6 months of diagnosis. | Mixed | Individual patient medical records. | No | 602 | 614 | 25 - 84 |
| CECILE Breast Cancer Study  (CECILE) | France | Population-based case-control study | All incident cases of breast cancer diagnosed in 2005-2007 among women <75 years of age and residing in Ille-et-Vilaine or Côte d'Or. Cases were recruited from the main cancer treatment center (Centre Eugène-Marquis in Rennes and Centre Georges-François-Leclerc in Dijon) and from private or public hospitals in each area. | Population-based case-control | Medical file and/or national file of death records | No | 0 | 859 | 25 - 74 |
| Copenhagen General Population Study  (CGPS) | Denmark | Population-based case-control study | Consecutive, incident cases from 1 hospital with centralized care for a population of 400,000 women from 2001 to the present. | Population-based case-control | Vital status: from the citizen registry | No | 4427 | 4492 | 24 - 98 |
| Cancer Prevention Study-II Nutrition Cohort  (CPSII) | USA | Nested case-control study | Breast cancer cases from the CPS-II Nutrition cohort were diagnosed between 1992 and 2009. The cases self-reported their diagnoses on biennial questionnaires and the diagnoses were then validated by medical records or state cancer registries. The participants in the Nutrition cohort were a subset of participants who lived in 21 U.S. states and were in the original CPS-II Cohort. The CPS-II Cohort participants were recruited in 1982 by American Cancer Society volunteers who went door-to-door to family, friends, and neighbors. | Nested case-control | Biennial questionnaires & National Death Index | No | 2411 | 2447 | 46 - 89 |
| California Teachers Study  (CTS) | USA | Prospective cohort study: nested case-control | This is a nested case-control study conducted within a cohort of California teachers(113,590) who were under age 80 years at baseline, had no prior history of invasive or in situ breast cancer. Cases are women newly diagnosed with a histologically confirmed invasive primary adenocarcinoma of the breast at age 80 years or younger from 1998 to 2008. | Nested case-control | The vital status and follow-up date are standard items of the California Cancer Registry | No | 0 | 1158 | 31 - 80 |
| DietCompLyf Breast Cancer Survival Study  (DIETCOMPLYF) | UK | Multi-centre prospective cohort study | Invasive primary breast cancer grade I-III, patients recruited 9 - 15 months after diagnosis, <age 75. Recruitment throughout UK. Patient first recruited on 18/2/97. Study joined NCRN in July 2004. Recruitment finished on 31/8/10 | Case-series | From the patients at the last time they visited the hospital. | No | 696 | 697 | 30 - 75 |
| European Prospective Investigation Into Cancer and Nutrition  (EPIC) | France, Germany, Greece, Italy, Spain, The Netherlands, and UK | Case-control study, nested in a prospective cohort study | Recruitment via 23 research centres in 10 European countries (Denmark, France, Germany, Greece, Italy, Norway, Spain, Sweden, The Netherlands, and UK) during the period 1992-2000. Cases are women diagnosed with invasive breast cancer after baseline. | Nested case-control | Direct contact or registry | No | 0 | 3358 | 29 - 88 |
| ESTHER Breast Cancer Study  (ESTHER) | Germany | Population-based case-control study | Statewide recruitment of breast cancer cases in all hospitals in Saarland/Germany in 2001-2003. | Population-based case-control | Residents' registration office and Saarland cancer registry | No | 0 | 468 | 30 - 79 |
| Family History Risk Study  (FHRISK) | UK | Clinic-based cohort study with a nested case-control study | Women diagnosed with breast cancer and attending the Family History Clinic in Manchester for increased risk of breast cancer. Recruitment period 2009-2012. | Mixed | Follow up continues until the participant has been discharged from the Family History Clinic | Yes | 144 | 146 | 29 - 78 |
| German Consortium for Hereditary Breast & Ovarian Cancer  (GC-HBOC) | Germany | Clinic-based case study and prospective cohort study | Women diagnosed with breast cancer in one of the GC-HBOC centres (Cologne, Munich, Kiel, Heidelberg, Düsseldorf, Ulm, Würzburg, Münster and Hannover). Recruitment period 1996-present. | Mixed | Medical records or personal visit for women under intensified surveillance | Yes | 1535 | 1629 | 19 - 86 |
| Gene Environment Interaction and Breast Cancer in Germany  (GENICA) | Germany | Population-based case-control study | Incident breast cancer cases enrolled between 2000 and 2004 from the Greater Bonn area (by of the hospitals within the study region); all enrolled within 6 months of diagnosis. | Population-based case-control | Through telephone interview with the patient or patient's relative, as well as information from the registration office and clinical records | No | 964 | 965 | 23 - 80 |
| Genetic Epidemiology Study of Breast Cancer by Age 50  (GESBC) | Germany | Population-based study of women <50 years | All incident cases diagnosed <50 years of age in 1992-5 in two regions: Rhein-Neckar-Odenwald and Freiburg, by surveying the 38 clinics serving these regions | Population-based case-control | Vital status was obtained by requesting this information from the population registry | No | 544 | 547 | 20 - 51 |
| Hannover Breast Cancer Study  (HABCS) | Germany | Hospital-based case-control study | Cases who received radiotherapy for breast cancer at Hannover Medical School between 1996-2003 (HaBCS I), or were diagnosed with breast cancer at a certified Breast Cancer Clinics in the Hannover region between 2012-2016 (HaBCS II), unselected for age or family history. | Mixed | Follow-up information was obtained through the central tumour registry at MHH; rarely through telephone contact with clinicians | No | 819 | 875 | 23 - 89 |
| Hospital Clinico San Carlos  (HCSC) | Spain | Population-based study of priori sporadic breast cancer cases | This is a cohort of a priori sporadic breast cancer patients which includes a cohort of 200 patients that were enrroled in a neoadyuvant trial in which women were randomized to neoadyuvant docetaxel vs neoadyuvant doxorrubicin. Recruitment period was from 2000 to 2013. Most patients have been treated in the Clinio San Carlos hospital (Madrid) and most and are being followed in different hospitals of Madrid. | Case-series | Medical records | No | 357 | 364 | 25 - 93 |
| Helsinki Breast Cancer Study  (HEBCS) | Finland | Hospital-based case-control study, plus additional familial cases | (1) Consecutive cases (883) from the Department of Oncology, Helsinki University Central Hospital 1997-8 and 2000, (2) Consecutive cases (986) from the Department of Surgery, Helsinki University Central Hospital 2001 – 2004, (3) Familial breast cancer patients (536) from the Helsinki University Central Hospital, Departments of Oncology and Clinical Genetics (1995-) | Mixed | Hospital medical records, Cancer registry, population registry | Subset (N=609) | 2118 | 2216 | 22 - 95 |
| Karolinska Breast Cancer Study  (KARBAC) | Sweden | Population and hospital-based cases; geographically matched controls | 1. Familial cases from Department of Clinical Genetics, Karolinska University Hospital , Stockholm. 2. Consecutive cases from Department of Oncology, Huddinge & Söder Hospital, Stockholm 1998-2000 | Mixed | Medical records | Subset (N=568) | 518 | 532 | 27 - 88 |
| Karolinska Mammography Project for Risk Prediction of Breast Cancer - Cohort Study  (KARMA) | Sweden | Cohort study | Inclusion of 70,877 women Oct 2010 - March 2013. 3000 women had BC at cohort entry. In all, 800 women have been diagnosed with breast cancer since study entry (Oct 2015). Approximately 250 women are diagnosed with BC annually | Nested case-control | Through the Swedish Cause of Death register, Clinical Breast Cancer register and the Inpatient register | No | 2578 | 2658 | 23 - 94 |
| Kuopio Breast Cancer Project  (KBCP) | Finland | Population-based prospective clinical cohort | 1. Women seen at Kuopio University Hospital between 1990 and 1995 because of breast lump, mammographic abnormality, or other breast symptom who were found to have breast cancer. 2. Consecutive malignant breast cancer cases diagnosed at KUH from 2011 onwards. | Population-based case-control | Follow-up is done by an oncologist | No | 435 | 440 | 23 - 91 |
| Kathleen Cuningham Foundation Consortium for research into Familial Breast Cancer/Australian Ovarian Cancer Study  (kConFab/AOCS) | Australia and New Zealand | Clinic-based recruitment of familial breast cancer patients (cases); population-based case-control study of ovarian cancer (controls only) | Cases were from multiple-case breast and breast-ovarian families recruited though family cancer clinics from across Australia and New Zealand from 1998 to the present. Cases were selected for inclusion in BCAC studies if (i) family was negative for mutations in BRCA1 and BRCA2 (ii) case was the index for the family, defined as youngest breast cancer affected family member. | Mixed | Patient self and family reports, medical records | Yes | 451 | 507 | 20 - 78 |
| Leuven Multidisciplinary Breast Centre  (LMBC) | Belgium | Hospital-based case-control study | All patients diagnosed with breast cancer and seen in the Multidisciplinary Breast Center in Leuven (Gashuisberg) since June 2007 plus retrospective collection of cases diagnosed since 2000 | Mixed | KWS; the latest data covers all departments, not only when they come for the breast pathology | No | 3138 | 3255 | 22 - 95 |
| Macedonian Breast Cancer Study  (MABCS) | Republic of North Macedonia | Hospital-based case-control study | Prospectively ascertained cases of breast cancer in two Hospitals in Skopje, Macedonia from 2012 to 2014. Ethnic origin: Macedonians (~82,8%) and Albanians (~17,2%). Age of the cases: 29 to 86, mean 53.8. | MIxed | No information available | No | 68 | 77 | 29 - 86 |
| Mammary Carcinoma Risk Factor Investigation  (MARIE) | Germany | Population-based case-control study | Incident cases diagnosed from 2001-2005 in the study region Hamburg in Northern Germany, and from 2002-2005 in the study region Rhein-Neckar-Karlsruhe in Southern Germany. | Population-based case-control | Follow-up information was obtained through follow-up interviews/questionnaires and new events through medical records to verify clinical events either reported by treating physicians or self-reported during follow-up interviews. Vital status was obtained by requesting this information from the population registry | No | 1534 | 1592 | 49 - 75 |
| Milan Breast Cancer Study Group  (MBCSG) | Italy | Clinic-based recruitment of familial/early onset breast cancer patients (cases); population-based controls | Familial and/or early onset breast cancer patients (aged 22-87) negative for mutations in BRCA genes, ascertained in two large cancer centres in Milan from 1996 to 2008 | Mixed | 80% Medical records; 5% Phone contact; 15% Referred by patients/family members | Yes | 412 | 473 | 18 - 76 |
| Mayo Clinic Breast Cancer Study  (MCBCS) | USA | Hospital-based case-control study | Incident cases residing in 6 states (MN, WI, IA, IL, ND, SD) seen at the Mayo Clinic in Rochester, MN from 2002-5 | Hospital-based case-control | Annual follow up surveys, medical records | No | 2032 | 2100 | 22 - 93 |
| Melbourne Collaborative Cohort Study  (MCCS) | Australia | Prospective cohort study: nested case-control study | Incident cases diagnosed between baseline (1990-1994) and last follow-up (2012) among the 24469 women participating in the cohort. | Nested case-control | Record linkage to the national and state cancer and death registries | No | 1191 | 1200 | 31 - 88 |
| Multiethnic Cohort  (MEC) | USA | Prospective cohort study: nested case-control | Incident cases identified from SEER cancer registries in Los Angeles County & State registries in California & Hawaii, USA from 1993-2002. Grouped by self-reported ethnicity. | Nested case-control | Linkage to SEER registries, state vital statistics and National Death Index | No | 703 | 718 | 46 - 90 |
| Melanoma Inquiry of Southern Sweden  (MISS) | Sweden | Population-based prospective cohort study | Population based cohort off women aged 25-65 in southern Sweden, born in Sweden, no cancer diagnosis before, interviewed about cancer risk factors 1990,2000,2010, saliva sampled 2011, cancer incidence/mortality followed through registries. | Nested case-control | Cause of death registry, records | No | 619 | 622 | 28 - 85 |
| Mayo Mammography Health Study  (MMHS) | USA | Prospective Cohort Study (2003-2006) of women ages 35+ receiving screening mammography at Mayo Clinic and living in MN, IA, WI; nested case-control | Incident cases (Invasive or in situ) diagnosed at least 3 months after enrollment | Nested case-control | Multiple sources: linkage to registration/mailings | No | 257 | 261 | 41 - 89 |
| Memorial Sloan-Kettering Cancer Center Study  (MSKCC) | USA | Case-control study | Incident and prevalent cases of histologically-confirmed breast cancer referred to the Clinical Genetics Service at MSKCC since July 1996. All cases tested negative for BRCA1/2 mutations. | Mixed |  | Yes | 509 | 529 | 21 - 85 |
| Norwegian Breast Cancer Study  (NBCS) | Norway | Hospital-based case-control study | Incidence cases from three different hospitals: 1) Cases (114) mean age 64 (28-92) at Ullevål Univ. Hospital 1990-94, 2) cases (182) mean age 59 (26-75) referred to Norwegian Radium Hospital 1975-1986, 3) cases (124), mean age 56 (29-82) with stage I or II disease, in the Oslo micro-metastases study at Norwegian Radium Hospital between 1995-1998, 4) Breast cancer cases referred to the Norwegian hospitals Akershus University Hospital in Lørenskog, Ullevaal university hospital in Oslo and Rikshospitalet-Radiumhospitalet in Oslo from 2007-2010. Mean age is 63 years. Consecutive series. 5) Breast cancer cases referred to the Norwegian Radium Hospital hospitalet 2010-2013. Neoadjuvantly treated with Avastin (Bevacizumab). 6) Consecutive series of Breast cancer incidents referred to Akershus university hospital 2004-2014. | Mixed | Medical records | No | 1959 | 1991 | 24 - 97 |
| Northern California Breast Cancer Family Registry  (NC-BCFR) | USA | Population-based recruitment of families; family-based cohort; population-based controls for subset of cases | Incident breast cancer cases included women aged <65 years diagnosed from 1995-2009, identified through the SEER cancer registry of the Greater San Francisco Bay Area. All cases with indicators of increased genetic risk were eligible to enroll (dx at age <35 yrs, personal history of ovarian or childhood cancer, bilateral breast cancer with 1st dx at age <50, family history of breast or ovarian cancer in first-degree relatives). Cases not meeting these criteria were randomly sampled (2.5% of non-Hispanic whites, 32% of other race/ethnicities). Incident cases also included men aged <80 years diagnosed from 1995-1998. | Mixed | Active follow-up by questionnaire and linkage with the California Cancer Registry. Annual phone follow-up from 1999-2012 to obtain updates on vital status and new cancers in the family. Updates on risk factors, vital status, and new cancers in family in 2007-2011, 2012-2014, and 2015-2017 | Subset (N=?) | 635 | 700 | 20 - 83 |
| North Carolina Breast Cancer Study  (NCBCS) | USA | NCBCS Phases 1 & 2: population-based case-control study.   NCBCS Phase 3: population-based case-only study.  In each phase, African American women and women under the age of 50 with invasive breast cancer were over-sampled. There was no over sampling by race or age for CIS cases in Phase 2. | NCBCS Phase 1: women aged of 20-74 residing in the 24 North Carolina county area and diagnosed with a first primary invasive breast cancer from 1993-1996.  NCBCS Phase 2: women aged of 20-74 residing in the same study area and diagnosed with a first primary invasive breast cancer from 1996-2000. All women diagnosed with DCIS, DCIS with microinvasion to a depth of 2mm, LCIS, and mixed of DCIS & LCIS from 1996-2000 were also eligible.  NCBCS Phase 3: Study area was expanded to 44 NC counties. Women aged 20-74 residing in this area and diagnosed with a first primary invasive breast cancer from May, 2008 to July, 2013. | Population-based case-control | Vital status was obtained through linkage to the US National Death Index. | No | 1988 | 2030 | 24 - 74 |
| Nurses' Health Study  (NHS) | USA | Prospective cohort study: nested case-control | Incident cases arising in the sub-cohort of 32,826 cohort members who gave a blood specimen in 1989-1990 are included if they were diagnosed with breast cancer prior to July 1, 2000. | Nested case-control | Follow up (recurrence): Biennial Questionnaire since 1976. Death: report from family member or post office or National Death Index. After death is registered, next-of-kin are contacted for additional information and for permission to access medical records. Information may also be sought from National Death Index, Tumor Registry or Death Certificate. | No | 0 | 1068 | 37 - 89 |
| Nurses' Health Study 2  (NHS2) | USA | Prospective cohort study: nested case-control | Incident cases arising in the sub-cohort of 29,611 women that donated blood during 1993 to 1995 are included. | Nested case-control | Follow up (recurrence): Biennial Questionnaire since 1989. Death: report from family member or post office or National Death Index. After death is registered, next-of-kin are contacted for additional information and for permission to access medical records. Information may also be sought from National Death Index, Tumor Registry or Death Certificate. | No | 0 | 1098 | 26 - 63 |
| Oulu Breast Cancer Study  (OBCS) | Finland | Hospital-based case-control study | Consecutive incident cases diagnosed at the Oulu University Hospital between 2000 and 2004. | Mixed | Hospital medical records, Cancer registry, population registry | No | 463 | 476 | 28 - 90 |
| Ontario Familial Breast Cancer Registry  (OFBCR) | Canada | Population-based familial case-control study | Cases diagnosed between 1 Jan 1996-31 Dec 1998 were identified from the Ontario Cancer Registry which registers >97% of all cases residing in the province at the time of diagnosis. All women with invasive breast cancer aged 20–54 years who met the OFBCR definition for high genetic risk (family history of specific cancers particularly breast and ovarian, early onset disease, Ashkenazi ethnicity or a diagnosis of multiple breast cancer) were asked to participate by completing risk factor questionnaires and providing a blood sample. A 25% random sample of individuals in this age category who did not meet the OFBCR definition, 35% of those aged 55–69 at high risk and 8.75% aged 55–69 at low risk were also asked to participate. Individuals diagnosed in 2001 and 2002 were also included if they met high -risk criteria. | Mixed | Follow-up data including vital status were collected though annual family history follow-up questionnires and though personal history follow-up questionnaires collected at years 10, 15 and 20 since baseline. Vital status of cases was ascertained also through linkage to cancer registry. | Subset (N=628) | 1873 | 1952 | 22 - 90 |
| Leiden University Medical Centre Breast Cancer Study  (ORIGO) | Netherlands | Hospital-based prospective cohort study | Consecutive cases diagnosed 1996-2006 in 2 hospitals of South-West Netherlands (Leiden & Rotterdam). No selection for family history; Rotterdam cases selected for diagnosis aged <70. Cases with in situ carcinomas eligible. | Mixed | Linkage to Municipal Population Register; National Pathology Registry; Hospital Information System; General Practitioner | No | 1172 | 1192 | 23 - 87 |
| NCI Polish Breast Cancer Study  (PBCS) | Poland | Population-based case-control study | Incident cases from 2000-2003 identified through a rapid identification system in participating hospitals covering ~ 90% of all eligible cases, and cancer registries in Warsaw and Łódź covering 100% of all eligible cases. | Population-based case-control | Complete follow-up for Warsaw cases; Only vital sttus for Łódz cases; We are reviewing medical records plus Cancer Registry data base plus Death Certificates data base | No | 1815 | 1841 | 27 - 75 |
| Karolinska Mammography Project for Risk Prediction of Breast Cancer - Case-Control Study  (pKARMA) | Sweden | Case-control study | Incident cases from Jan 2001 – Dec 2008 from the Stockholm/Gotland area. Identified through the Stockholm breast cancer registry. | Mixed | Through the Swedish Cause of Death register, Clinical Breast Cancer register and the Inpatient register | No | 4844 | 5014 | 25 - 86 |
| The Prostate,Lung,Colorectal and Ovarian Cancer Screening Trial  (PLCO) | USA | Prospective cohort study: nested case-control | Incident cases arising in the sub-cohort of 78,232 women who gave a blood specimen in 1993-2001 are included if they were diagnosed with breast cancer. Recruitment via mutiple screening centers across the US. | Nested case-control | Vital status information is collected through annual follow-up that attempts to contact participants. A National Death Index search was conducted annually for participants who had not responded to repeated attempts to contact them | No | 0 | 1797 | 55 - 88 |
| Prospective Study of Outcomes in Sporadic Versus Hereditary Breast Cancer  (POSH) | UK | Prospective cohort | Cases aged 40 or younger at breast cancer diagnosis. Recruited from breast cancer centre oncology clinics across 126 UK hospitals and diagnosed between January 2000 to December 2007. | Case-series | National data | No | 1079 | 1081 | 18 - 41 |
| Evaluation of Predictive Factors regarding the Effectivity of Aromatase Inhibitor Therapy  (PREFACE) | Germany | Multicenter, prospective, randomized, open-label phase IV study | Postmenopausal, steroid hormone receptor positive breast cancer patients who are treated with letrozole. Recruitment at multicentres in Germany between 2009-2011. | Case-series | Medical records | No | 2394 | 2476 | 41 - 95 |
| Predicting the Risk Of Cancer At Screening Study  (PROCAS) | UK | Population based study | Women diagnosed with breast cancer since joining the study of women attending the Breast Screening Programme (NHSBSP) in Greater Manchester. Recruitment period Oct 2009-May 2014. | Population-based case-control | Cancer report updated monthly from NHS systems. Deaths report updated weekly from local GP records | No | 375 | 380 | 45 - 76 |
| Rotterdam Breast Cancer Study  (RBCS) | Netherlands | Hospital-based case-control study, Rotterdam area | Familial breast cancer patients selected from the Clinical Genetics Center at Erasmus MC Cancer Institute; recruited 1994 - 2005 (RBCS1) and 1995 - 2009 (RBCS2; for OncoArray). | Mixed | From medical file/ info from GP or other hospital. Vital status also from Municipal registry | Yes | 1010 | 1049 | 18 - 84 |
| Singapore and Sweden Breast Cancer Study  (SASBAC) | Sweden | Population-based case-control study | Incident cases from October 1993 to March 1995 identified via the 6 regional cancer registries in Sweden, to which reporting is mandatory. | Population-based case-control | Through medical records the first 6-8 years and the Swedish Cause of Death register, Clinical Breast Cancer register and the Inpatient register there after | No | 1186 | 1203 | 50 - 75 |
| Sheffield Breast Cancer Study  (SBCS) | UK | Hospital-based case-control study | Women with pathologically confirmed breast cancer recruited from surgical outpatient clinics at the Royal Hallamshire Hospital, Sheffield, 1998 – 2005; cases are a mixture of prevalent and incident disease | Mixed | Trent Cancer Registry | No | 936 | 955 | 24 - 92 |
| Study of Epidemiology and Risk factors in Cancer Heredity  (SEARCH) | UK | Population-based case-control study | 2 groups of cases identified through East Anglian Cancer Registry; 1) prevalent cases diagnosed 1991-1996 under 55 years of age at diagnosis, recruited 1996-2002; 2) incident cases diagnosed since 1996 under 70 years of age at diagnosis, recruited 1996-present. | Mixed | The National Cancer Registration and Analysis Service | No | 12860 | 12927 | 21 - 88 |
| Städtisches Klinikum Karlsruhe Deutsches Krebsforschungszentrum Study  (SKKDKFZS) | Germany | Hospital-based breast cancer cohort | Women diagnosed with primary *in situ* or invasive breast cancer at the Städtisches Klinikum Karlsruhe from March 1993 to July 2005. | Case-series | Follow-up (including vital status) information was obtained from medical records, pathology reports or population registries. Risk factor data were collected from about 10% of patients by questionnaire; from the remaining patients risk factor data were obtained from the medical records. | No | 1096 | 1137 | 21 - 93 |
| Swedish Mammography Cohort  (SMC) | Sweden | Nested case control study from a population-based cohort | All breast cancer cases in the cohort (information from the Swedish Cancer Register) from 1987-2011 for women who gave saliva in 2005-2008 or a blood specimen in 2003-2009 are included. | Nested case-control | Swedish Death Register & Swedish Cancer Register | No | 0 | 1487 | 41 - 91 |
| Simultaneous Study of Gemcitabine-Docetaxel Combination adjuvant treatment  (SUCCESSB) | Germany | Multicenter, prospective, randomized, open-label phase III study | Patients with primary Her2-positive and high risk breast cancer (pN+ oder >pT1b or >G1 or <36y or HR-). Recruitment at multicentres in Germany during 2008-2011. | Case-series | Medical records | No | 435 | 436 | 28 - 81 |
| Simultaneous Study of Docetaxel Based Anthracycline Free Adjuvant Treatment Evaluation  (SUCCESSC) | Germany | Multicenter, prospective, randomized, open-label phase III study | Patients with primary Her2-negative and high risk breast cancer (pN+ oder >pT1b or >G1 or <36y or HR-). Recruitment at multicentres in Germany during 2009-2011. | Case-series | Medical records | No | 2786 | 2800 | 24 - 79 |
| IHCC-Szczecin Breast Cancer Study  (SZBCS) | Poland | Hospital-based case-control study | Prospectively ascertained cases of invasive breast cancer patients diagnosed at the Regional Oncology Hospital (Szczecin) in the years 2002, 2003, 2006 and 2007 or the University Hospital from 2002 to 2007 in Szczecin, West-Pomerania, Poland. Patients with pure intraductal or intralobular cancer were excluded (DCIS or LCIS) but patients with DCIS with micro-invasion were included. | Mixed | Questionnaire sent by post | No | 957 | 1002 | 25 - 88 |
| Utah Breast Cancer Study  (UBCS) | USA | Mixed. (1) Pedigrees including multiple sampled breast cancer cases within 2 generations, also may include sampled, unaffected relatives; (2) hospital-based cases (from Huntmans Cancer Institute [HCI] or Intermountain Healthcare [IH]), and breast reduction controls; and (3) Population-based cases (from the Utah Cancer Registry [UCR]) and controls (from the Utah Drivers License Registry [UDLR]) | Cases recruited from late 1970s to present (on-going). Ascertainment from: (1) UCR-confirmed breast cancer cases in high-risk pedigrees; (2) invasive breast cancer cases treated or surgery performed at HCI or IH clinics; (3) prevalent, population-based UCR-confirmed breast cancer cases. | Mixed | Utah Population Database (UPDB). Events captured by the database include: death record, marriage record, birth record, cancer diagnoses (all cancers), census data, hospital visits, drivers license renewals, etc. Last event in the UPDB via these state/hospital records indicate living/death status. | Subset (N=250) | 532 | 558 | 28 - 92 |
| UCI Breast Cancer Study  (UCIBCS) | USA | Population-based case-control study | All cases diagnosed in Orange County, California, during one-year period beginning March 1, 1994. Ascertained through the population-based Cancer Surveillance Program of Orange County California (CSPOC) | Mixed | The vital status and follow-up date are standard items of the California Cancer Registry | No | 752 | 752 | 24 - 90 |
| UK Breakthrough Generations Study  (UKBGS) | UK | Nested case-control study of incident and prevalent cases within prospective cohort study | Depending on the BCAC analysis, either cohort members who had breast cancer before entry into the Breakthrough Generations Study (cohort of 100,000+ women followed up for breast cancer, recruited from the UK during 2003-2011), or members who developed breast cancer during follow-up. | Nested case-control | Follow-up questionnaires and national data systems | No | 3250 | 3252 | 24 - 87 |
| US Three State Study  (US3SS) | USA | Population-based case-control study | Eligible cases were all English-speaking female residents of Massachusetts (excluding metropolitan Boston), New Hampshire and Wisconsin, with a new diagnosis of invasive (aged 20–69 years) or in situ breast cancer (aged 20–74 years, MA and NH only) reported to each state's mandatory cancer registry during 1998-2001. | Population-based case-control |  | No | 0 | 1206 | 28 - 69 |
| US Radiologic Technologists Study  (USRT) | USA | Population -based case-control study using prevalent cases with controls matched to cases on year of birth | Prevalent cases identified through mailed surveys in 1983-8 and 1994-8, incident cases between surveys; blood collected from 1999-2004; unselected for family cancer history or any other characteristics; most cases sampled more than 5 years after diagnosis | Mixed | Cancer incidence is obtained from follow-up, mailed questionnaires. Vital status follow-up was coordinated with the ARRT’s registration renewal cycle, which is conducted annually by birth month. The monthly certification renewal files received from ARRT provide an on-going mechanism for updating vital status and addresses for cohort members who are actively registered by the ARRT. Various tracing resources were used over time to obtain current addresses or death notifications for inactive registrants, including post office address correction requests, telephone and other directories, credit bureau reports, motor vehicle department records, state mortality and other files, and linkages with Social Security Administration, National Death Index (NDI), and other databases. | No | 0 | 1266 | 26 - 87 |
| Total |  |  |  |  |  |  | 82701 | 100973 |  |

* Among the cases included for multiple imputation
