# Supplementary material for: Association of the CHEK2 c.1100delC variant, radiotherapy, and systemic treatment with contralateral breast cancer risk and breast cancer‐specific survival
Source: Cancer Med. 2023 Jul 3;12(15):16142–62. doi: 10.1002/cam4.6272 (PMC10469654; doi:10.1002/cam4.6272)
Supplement: Supplementary file 3 — Tables S2–S18. [file CAM4-12-16142-s003.docx]

**Table S2.** Overview of the variables included for multiple imputation with the R package MICE.

| **Variable** | **Missing data**  **Percentage*** | **Pre-processing performed before imputation** | **Imputation method** |
| --- | --- | --- | --- |
| Year of diagnosis | 4.1 |  | Predictive mean matching |
| Ethnicity class | 9.5 |  | Polytomous regression |
| Morphology group of the first BC | 14.4 |  | Polytomous regression |
| ER status of the first BC | 18.4 |  | Logistic regression |
| Lymph node status of the first BC | 21.9 |  | Logistic regression |
| Histopathological grade of the first BC | 22.6 |  | Polytomous regression |
| CBC status** | 27.3 |  | Predictive mean matching |
| Time to CBC | 27.4 |  | Predictive mean matching |
| Size category of the first BC | 28.3 |  | Polytomous regression |
| Number of positive lymph nodes for the first BC | 28.5 |  | Predictive mean matching |
| Behavior of the second BC | 28.6 |  | Polytomous regression |
| Morphology group of the second BC | 29.1 |  | Polytomous regression |
| PR status of the first BC | 29.4 |  | Logistic regression |
| Histopathological grade of the second BC | 29.7 |  | Polytomous regression |
| Lymph node status of the second BC | 29.8 |  | Polytomous regression |
| ER status of the second BC | 29.8 |  | Polytomous regression |
| PR status of the second BC | 29.9 |  | Polytomous regression |
| Size category of the second BC | 29.9 |  | Polytomous regression |
| Distant metastases status of the second BC | 30.3 |  | Polytomous regression |
| HER2 status of the second BC | 30.5 |  | Polytomous regression |
| Size of the first BC in mm | 33.9 |  | Predictive mean matching |
| Tumor stage of the first BC | 38.6 |  | Polytomous regression |
| Surgery | 42.1 |  | Polytomous regression |
| Adjuvant CT | 42.2 |  | Logistic regression |
| Adjuvant ET | 46.3 |  | Logistic regression |
| Radiotherapy | 46.3 |  | Polytomous regression |
| HER2 status of the first BC | 47.6 |  | Logistic regression |
| Neo-adjuvant CT | 50.5 |  | Logistic regression |
| Anthracyclines (neo-adjuvant) | 51.7 |  | Logistic regression |
| Taxanes (neo-adjuvant) | 51.7 |  | Logistic regression |
| CMF-like CT (neo-adjuvant) | 51.8 |  | Logistic regression |
| CMF-like CT (adjuvant) | 52.2 |  | Logistic regression |
| Taxanes (adjuvant) | 52.3 |  | Logistic regression |
| Anthracyclines (adjuvant) | 52.6 |  | Logistic regression |
| Trastuzumab | 57.2 | If missing and corresponding value of Year of diagnosis observed and < 1998, then set equal to 0 (=no trastuzumab). | Logistic regression |
| Aromatase inhibitor | 57.4 |  | Logistic regression |
| Tamoxifen | 57.4 |  | Logistic regression |

Abbreviations: BC=breast cancer; CBC=contralateral breast cancer; ER=estrogen receptor; PR=progesterone receptor; HER2=human epidermal growth factor receptor 2; CT=chemotherapy; ET=endocrine therapy; CMF Cyclophosphamide Methotrexate Fluorouracil. *Based on the total number of patients included in the imputation process (100,973) as specified in Table S1.**Event indicator for CBC. Note: The Nelson-Aalen estimator of the baseline cumulative hazard and the event indicator of overall survival and breast cancer-specific survival were included in all imputation models to improve imputation, as well as the time to contralateral breast cancer and the corresponding event indicator. Imputed values of CBC status and time to CBC were not used in the analyses.

**Table S3.** Tumor characteristics for the CBC by *CHEK2* c.1100delC carrier status.

| **Characteristics** | **Non- carriers** | ***CHEK2* c.1100delC carriers** | **P-value** |
| --- | --- | --- | --- |
| **Number of CBC occurrences, n** | 1,757 | 59 |  |
| Year of primary diagnosis, median (IQR) | 2000 (1996-2003) | 1998 (1996-2000) | 0.04 |
| Year of CBC diagnosis, median (IQR) | 2007 (2002-2011) | 2003 (2000-2009) | 0.002 |
| Age at primary diagnosis, y, median (IQR) | 52 (43-62) | 48 (38-56) | 0.01 |
| Age at CBC diagnosis, y, median (IQR) | 60 (51-69) | 52 (45-62) | < 0.001 |
| **Tumor characteristics** |  |  |  |
| **Tumor size**, n (%)  ≤2 cm  >2 and ≤5 cm  >5 cm  Missing, n | 444 (73.8)  130 (21.6)  28 (4.7)  1,155 | 19 (76.0)  6 (24.0)  0 (0.0)  34 | 0.54 |
| **Behavior**, n (%)  Invasive  In-situ  Missing, n | 1,048 (86.3)  166 (13.7)  543 | 42 (80.8)  10 (19.2)  7 | 0.35 |
| **Lymph node status**, n (%)  Negative  Positive  Missing, n | 448 (70.0)  192 (30.0)  1,117 | 15 (55.6)  12 (44.4)  32 | 0.02 |
| **Grade**, n (%)  Grade 1  Grade 2  Grade 3  Missing, n | 126 (17.8)  318 (45.0)  263 (37.2)  1,050 | 5 (15.6)  18 (56.3)  9 (28.1)  27 | 0.44 |
| **Morphology**, n (%)  Ductal  Lobular  Medullary  Mixed (ductal & lobular)  Mucinous  Papillary  Tubular  Other  Missing, n | 625 (67.7)  144 (15.6)  6 (0.7)  57 (6.2)  7 (0.8)  1 (0.1)  13 (1.4)  70 (7.6)  834 | 24 (60.0)  9 (22.5)  0 (0.0)  4 (10.0)  0 (0.0)  0 (0.0)  0 (0.0)  3 (7.5)  19 | 0.83 |
| **ER status**, n (%)  Negative  Positive  Missing, n | 168 (25.0)  503 (75.0)  1,086 | 6 (23.1)  20 (76.9)  33 | 1.00 |
| **PR status**, n (%)  Negative  Positive  Missing, n | 255 (39.8)  386 (60.2)  1,116 | 8 (30.8)  18 (69.2)  33 | 0.47 |
| **HER2 status**, n (%)  Negative  Positive  Missing, n | 348 (84.5)  64 (15.5)  1,345 | 13 (86.7)  2 (13.3)  44 | 1.00 |

Percentages are only on observed, non-missing data, and may not total 100 because of rounding. Abbreviations: CBC=contralateral breast cancer; ER=estrogen receptor; PR=progesterone receptor; HER2=human epidermal growth factor receptor 2.

**Table S4:** Contralateral breast cancer risk (hazard ratio) by treatment for first primary breast cancer and *CHEK2* c.1100delC mutation status. Stratified by time since first primary breast cancer diagnosis. Sensitivity analysis in patients aged 40 years and younger at time of first primary breast cancer diagnosis.

|  | | **Total follow-up time** | | | **< 5-year follow-up** | | | **> 5 years follow-up** | | |
| --- | --- | --- | --- | --- | --- | --- | --- | --- | --- | --- |
| No of patients | | 9,642 | | | 8,307 | | | 7,292 | | |
| No of CBC events | | 345 | | | 130 | | | 215 | | |
|  | | HR (95%CI) | P-value | P-int | HR (95%CI) | P-value | P-int | HR (95%CI) | P-value | P-int |
| *CHEK2* c.1100delC status | | 3.24 (2.02-5.18) | <0.001 |  | 4.28 (2.22-8.25) | <0.001 |  | 2.56 (1.29-5.11) | 0.01 |  |
| Radiotherapy | |  |  | 0.23 |  |  | 0.48 |  |  | 0.31 |
|  | No radiotherapy | ref |  |  | ref |  |  | ref |  |  |
|  | Radiotherapy | 1.18 (0.88-1.58) | 0.26 |  | 1.17 (0.70-1.94) | 0.54 |  | 1.18 (0.83-1.69) | 0.35 |  |
| Systemic therapy | |  |  | 0.66 |  |  | 0.97 |  |  | 0.43 |
|  | No systemic therapy | ref |  |  | ref |  |  | ref |  |  |
|  | CT, no ET | 0.70 (0.48-1.03) | 0.07 |  | 0.54 (0.28-1.05) | 0.07 |  | 0.81 (0.50-1.31) | 0.40 |  |
|  | ET, no CT | 0.76 (0.48-1.22) | 0.26 |  | 0.54 (0.22-1.31) | 0.17 |  | 0.88 (0.50-1.55) | 0.65 |  |
|  | Both CT and ET | 0.58 (0.39-0.86) | 0.01 |  | 0.42 (0.22-0.80) | 0.01 |  | 0.69 (0.42-1.12) | 0.13 |  |

Adjusted for age at diagnosis, ER-status, nodal status, size category and grade of primary BC. Abbreviations: CT=chemotherapy; ET=endocrine therapy; P-int= P-value for the comparison of a model including an interaction term between *CHEK2* c.1100delC status and a specific treatment (radiotherapy or systemic treatment) with a model without any interaction term.

**Table S5:** Contralateral breast cancer risk (hazard ratio) by treatment for primary BC and *CHEK2* c.1100delC mutation status. Stratified by time since breast cancer diagnosis. Sensitivity analysis restricting to patients diagnosed from the year 2000 onwards

|  | | **Total follow-up time** | | | **< 5-year follow-up** | | | **> 5 years follow-up** | | |
| --- | --- | --- | --- | --- | --- | --- | --- | --- | --- | --- |
| No of patients | | 59,296 | | | 55,349 | | | 41,952 | | |
| No of CBC events | | 910 | | | 394 | | | 516 | | |
|  | | HR (95%CI) | P-value | P-int | HR (95%CI) | P-value | P-int | HR (95%CI) | P-value | P-int |
| *CHEK2* c.1100delC status | | 1.85 (1.15-2.97) | 0.01 |  | 1.66 (0.81-3.37) | 0.16 |  | 2.06 (1.09-3.89) | 0.03 |  |
| Radiotherapy | |  |  | 0.80 |  |  | 0.90 |  |  | 0.84 |
|  | No radiotherapy | ref |  |  | ref |  |  | ref |  |  |
|  | Radiotherapy | 0.99 (0.83-1.18) | 0.92 |  | 0.90 (0.70-1.16) | 0.42 |  | 1.07 (0.85-1.35) | 0.56 |  |
| Systemic therapy | |  |  | 1.00 |  |  | 0.99 |  |  | 0.95 |
|  | No systemic therapy | ref |  |  | ref |  |  | ref |  |  |
|  | CT, no ET | 0.75 (0.52-1.09) | 0.13 |  | 0.53 (0.31-0.89) | 0.02 |  | 0.98 (0.60-1.59) | 0.93 |  |
|  | ET, no CT | 0.69 (0.52-0.91) | 0.01 |  | 0.59 (0.38-0.92) | 0.02 |  | 0.75 (0.53-1.07) | 0.12 |  |
|  | Both CT and ET | 0.67 (0.50-0.90) | 0.01 |  | 0.50 (0.32-0.79) | 0.003 |  | 0.83 (0.571.20) | 0.32 |  |

Adjusted for age at diagnosis, ER-status, nodal status, size category and grade of primary BC. Abbreviations: CT=chemotherapy; ET=endocrine therapy; P-int= P-value for the comparison of a model including an interaction term between *CHEK2* c.1100delC status and a specific treatment (radiotherapy or systemic treatment) with a model without any interaction term.

**Table S6:** Contralateral breast cancer risk (hazard ratio) by treatment for primary BC and *CHEK2* c.1100delC mutation status. Stratified by time since breast cancer diagnosis (complete-case analysis).

|  | | **Total follow-up time** | | | **< 5-year follow-up** | | | **> 5 years follow-up** | | |
| --- | --- | --- | --- | --- | --- | --- | --- | --- | --- | --- |
| No of patients | | 29,842 | | | 28,443 | | | 24,265 | | |
| No of CBC events | | 703 | | | 274 | | | 429 | | |
|  | | HR (95%CI) | P-value | P-int | HR (95%CI) | P-value | P-int | HR (95%CI) | P-value | P-int |
| *CHEK2* c.1100delC status | | 1.86 (1.17-2.94) | 0.01 |  | 3.14 (1.78-5.54) | <0.001 |  | 1.00 (0.452.25) | 1.00 |  |
| Radiotherapy | |  |  | 0.78 |  |  | 0.26 |  |  | 0.05* |
|  | No radiotherapy | ref |  |  | ref |  |  | ref |  |  |
|  | Radiotherapy | 1.10 (0.90-1.33) | 0.35 |  | 1.07 (0.79-1.44) | 0.68 |  | 1.12 (0.87-1.44) | 0.36 |  |
| Systemic therapy | |  |  | 0.71 |  |  | 0.89 |  |  | 0.18 |
|  | No systemic therapy | ref |  |  | ref |  |  | ref |  |  |
|  | CT, no ET | 0.72 (0.54-0.96) | 0.03 |  | 0.46 (0.29-0.73) | 0.001 |  | 0.96 (0.67-1.38) | 0.83 |  |
|  | ET, no CT | 0.73 (0.55-0.96) | 0.02 |  | 0.60 (0.39-0.93) | 0.02 |  | 0.82 (0.58-1.17) | 0.28 |  |
|  | Both CT and ET | 0.72 (0.54-0.96) | 0.03 |  | 0.42 (0.27-0.67) | <0.001 |  | 1.03 (0.71-1.49) | 0.88 |  |

Adjusted for age at diagnosis, ER-status, nodal status, size category and grade of primary BC. Abbreviations: CT=chemotherapy; ET=endocrine therapy; P-int= P-value for the comparison of a model including an interaction term between *CHEK2* c.1100delC status and a specific treatment (radiotherapy or systemic treatment) with a model without any interaction term. * The corresponding complete model including the interaction term between *CHEK2* c.1100delC status and radiotherapy is shown in Table S10.

**Table S7:** Contralateral breast cancer risk (hazard ratio) by treatment for primary BC and *CHEK2* c.1100delC mutation status. Stratified by time since breast cancer diagnosis. Sensitivity analysis in patients aged 40 years and younger at time of first BC diagnosis (complete–case analysis).

|  | | **Total follow-up time** | | | **< 5-year follow-up** | | | **> 5 years follow-up** | | |
| --- | --- | --- | --- | --- | --- | --- | --- | --- | --- | --- |
| No of patients | | 3,665 | | | 3,473 | | | 2,935 | | |
| No of CBC events | | 129 | | | 53 | | | 76 | | |
|  | | HR (95%CI) | P-value | P-int | HR (95%CI) | P-value | P-int | HR (95%CI) | P-value | P-int |
| *CHEK2* c.1100delC status | | 2.73 (1.18-6.30) | 0.02 |  | 2.68 (0.81-8.93) | 0.11 |  | 2.54 (0.78-8.23) | 0.12 |  |
| Radiotherapy | |  |  | 0.68 |  |  | 0.21 |  |  | 0.21 |
|  | No radiotherapy | ref |  |  | ref |  |  | ref |  |  |
|  | Radiotherapy | 1.58 (0.96-2.59) | 0.07 |  | 1.07 (0.52-2.19) | 0.86 |  | 2.12 (1.06-4.22) | 0.03 |  |
| Systemic therapy | |  |  | 0.10* |  |  | 0.43 |  |  | 0.27 |
|  | No systemic therapy | ref |  |  | ref |  |  | ref |  |  |
|  | CT, no ET | 0.89 (0.47-1.70) | 0.73 |  | 0.41 (0.15-1.13) | 0.09 |  | 1.56 (0.68-3.60) | 0.29 |  |
|  | ET, no CT | 1.01 (0.41-2.49) | 0.98 |  | 0.83 (0.21-3.30) | 0.79 |  | 1.25 (0.38-4.11) | 0.71 |  |
|  | Both CT and ET | 0.68 (0.34-1.34) | 0.26 |  | 0.26 (0.09-0.76) | 0.01 |  | 1.36 (0.56-3.29) | 0.49 |  |

Adjusted for age at diagnosis, ER-status, nodal status, size category and grade of primary BC. Abbreviations: CT=chemotherapy; ET=endocrine therapy; P-int= P-value for the comparison of a model including an interaction term between *CHEK2* c.1100delC status and a specific treatment (radiotherapy or systemic treatment) with a model without any interaction term. * The corresponding complete model including the interaction term between *CHEK2* c.1100delC status and systemic therapy is shown in Table S11.

**Table S8:** Contralateral breast cancer risk (hazard ratio) by treatment for primary BC and *CHEK2* c.1100delC mutation status. Stratified by time since breast cancer diagnosis. Sensitivity analysis restricting to patients diagnosed with a ER-positive first breast cancer (complete–case analysis).

|  | | **Total follow-up time** | | | **< 5-year follow-up** | | | **> 5 years follow-up** | | |
| --- | --- | --- | --- | --- | --- | --- | --- | --- | --- | --- |
| No of patients | | 23,924 | | | 22,781 | | | 19,775 | | |
| No of CBC events | | 525 | | | 202 | | | 323 | | |
|  | | HR (95%CI) | P-value | P-int | HR (95%CI) | P-value | P-int | HR (95%CI) | P-value | P-int |
| *CHEK2* c.1100delC status | | 2.19 (1.36-3.52) | 0.001 |  | 3.94 (2.21-7.02) | <0.001 |  | 1.02 (0.42-2.48) | 0.97 |  |
| Radiotherapy | |  |  | 0.68 |  |  | 0.25 |  |  | 0.07* |
|  | No radiotherapy | ref |  |  | ref |  |  | ref |  |  |
|  | Radiotherapy | 1.05 (0.84-1.31) | 0.68 |  | 1.04 (0.73-1.48) | 0.83 |  | 1.06 (0.80-1.41) | 0.68 |  |
| Systemic therapy | |  |  | 0.74 |  |  | 0.96 |  |  | 0.22 |
|  | No systemic therapy | ref |  |  | ref |  |  | ref |  |  |
|  | CT, no ET | 0.71 (0.48-1.05) | 0.09 |  | 0.55 (0.28-1.05) | 0.07 |  | 0.83 (0.51-1.37) | 0.48 |  |
|  | ET, no CT | 0.67 (0.49-0.92) | 0.01 |  | 0.54 (0.32-0.89) | 0.02 |  | 0.75 (0.50-1.12) | 0.16 |  |
|  | Both CT and ET | 0.68 (0.48-0.94) | 0.02 |  | 0.40 (0.23-0.69) | <0.001 |  | 0.93 (0.61-1.42) | 0.73 |  |

Adjusted for age at diagnosis, ER-status, nodal status, size category and grade of primary BC. Abbreviations: CT=chemotherapy; ET=endocrine therapy; P-int= P-value for the comparison of a model including an interaction term between *CHEK2* c.1100delC status and a specific treatment (radiotherapy or systemic treatment) with a model without any interaction term.* The corresponding complete model including the interaction term between *CHEK2* c.1100delC status and radiotherapy is shown in Table S12.

**Table S9:** Contralateral breast cancer risk (hazard ratio) by treatment for primary BC and *CHEK2* c.1100delC mutation status. Stratified by time since breast cancer diagnosis. Sensitivity analysis restricting to patients diagnosed from the year 2000 onwards (complete–case analysis).

|  | | **Total follow-up time** | | | **< 5-year follow-up** | | | **> 5 years follow-up** | | |
| --- | --- | --- | --- | --- | --- | --- | --- | --- | --- | --- |
| No of patients | | 23,277 | | | 22,483 | | | 18,558 | | |
| No of CBC events | | 425 | | | 176 | | | 249 | | |
|  | | HR (95%CI) | P-value | P-int | HR (95%CI) | P-value | P-int | HR (95%CI) | P-value | P-int |
| *CHEK2* c.1100delC status | | 1.47 (0.72-2.98) | 0.29 |  | 2.49 (1.08-5.74) | 0.03 |  | 0.66 (0.16-2.67) | 0.56 |  |
| Radiotherapy | |  |  | 0.42 |  |  | 1.00 |  |  | 0.14 |
|  | No radiotherapy | ref |  |  | ref |  |  | ref |  |  |
|  | Radiotherapy | 0.99 (0.77-1.27) | 0.94 |  | 1.04 (0.71-1.54) | 0.84 |  | 0.95 (0.69-1.31) | 0.76 |  |
| Systemic therapy | |  |  | 0.55 |  |  | 0.66 |  |  | 0.44 |
|  | No systemic therapy | ref |  |  | ref |  |  | ref |  |  |
|  | CT, no ET | 0.55 (0.35-0.85) | 0.01 |  | 0.40 (0.21-0.78) | 0.01 |  | 0.68 (0.37-1.23) | 0.20 |  |
|  | ET, no CT | 0.63 (0.44-0.91) | 0.01 |  | 0.56 (0.31-1.00) | 0.05 |  | 0.68 (0.42-1.10) | 0.12 |  |
|  | Both CT and ET | 0.67 (0.46-0.98) | 0.04 |  | 0.46 (0.26-0.84) | 0.01 |  | 0.87 (0.53-1.44) | 0.60 |  |

Adjusted for age at diagnosis, ER-status, nodal status, size category and grade of primary BC. Abbreviations: CT=chemotherapy; ET=endocrine therapy; P-int= P-value for the comparison of a model including an interaction term between *CHEK2* c.1100delC status and a specific treatment (radiotherapy or systemic treatment) with a model without any interaction term.

**Table S10:** Output of the full model for the complete-case analysis with follow-up starting from 5 years after the diagnosis of the first breast cancer, including an interaction term between *CHEK2* c.1100delC status and radiotherapy.

|  | | **> 5 years follow-up** | |
| --- | --- | --- | --- |
| No of patients, non-*CHEK2* | | 23,991 | |
| No of CBC events, non-*CHEK2* | | 423 | |
| No of patients, *CHEK2* | | 274 | |
| No of CBC events, *CHEK2* | | 6 | |
|  | |  | |
|  | | HR (95%CI) | P-value |
| *CHEK2* c.1100delC status | | 8.7E-07 (0.00-INF) | 0.99 |
| Systemic therapy | |  |  |
|  | No systemic therapy | ref |  |
|  | CT, no ET | 0.96 (0.67-1.38) | 0.82 |
|  | ET, no CT | 0.82 (0.58-1.17) | 0.27 |
|  | Both CT and ET | 1.03 (0.71-1.48) | 0.89 |
| Radiotherapy | |  |  |
|  | No radiotherapy | ref |  |
|  | Radiotherapy | 1.09 (0.85-1.41) | 0.48 |
| Age at diagnosis | | 1.00 (0.99-1.01) | 0.45 |
| ER-status of first BC | |  |  |
|  | Negative | ref |  |
|  | Positive | 0.96 (0.73-1.27) | 0.77 |
| Grade of first BC | |  |  |
|  | Grade 1 | Ref |  |
|  | Grade 2 | 1.00 (0.77-1.32) | 0.97 |
|  | Grade 3 | 1.19 (0.88-1.59) | 0.26 |
| Nodal status of first BC | |  |  |
|  | Negative | Ref |  |
|  | Positive | 0.94 (0.74-1.18) | 0.58 |
| Size category of first BC | |  |  |
|  | 0-2 cm | Ref |  |
|  | 2-5 cm | 0.88 (0.71-1.09) | 0.24 |
|  | >5 cm | 0.63 (0.34-1.16) | 0.14 |
|  |  |  |  |
|  | CHEK2:RT | 1.6E+06 (0.00-INF) | 0.99 |

Adjusted for age at diagnosis, ER-status, nodal status, size category and grade of primary BC. Abbreviations: CT=chemotherapy; ET=endocrine therapy; RT: radiotherapy.

**Table S11:** Output of the full model for the complete-case analysis restricted to patients aged 40 years and younger at time of first BC diagnosis, over the total follow-up time, including an interaction term between *CHEK2* c.1100delC status and systemic therapy.

|  | | **Total FU** | |
| --- | --- | --- | --- |
| No of patients, non-*CHEK2* | | 3,599 | |
| No of CBC events, non-*CHEK2* | | 123 | |
| No of patients, *CHEK2* | | 66 | |
| No of CBC events, *CHEK2* | | 6 | |
|  | |  | |
|  | | HR (95%CI) | P-value |
| *CHEK2* c.1100delC status | | 2.3E-07 (0.00-INF) | 1.00 |
| Systemic therapy | |  |  |
|  | No systemic therapy | Ref |  |
|  | CT, no ET | 0.77 (0.40-1.48) | 0.44 |
|  | ET, no CT | 1.00 (0.41-2.47) | 1.00 |
|  | Both CT and ET | 0.63 (0.32-1.24) | 0.18 |
| Radiotherapy | |  |  |
|  | No radiotherapy | Ref |  |
|  | Radiotherapy | 1.60 (0.97-2.63) | 0.07 |
| Age at diagnosis | | 0.97 (0.93-1.02) | 0.26 |
| ER-status of first BC | |  |  |
|  | Negative | Ref |  |
|  | Positive | 1.35 (0.85-2.15) | 0.20 |
| Grade of first BC | |  |  |
|  | Grade 1 | Ref |  |
|  | Grade 2 | 2.52 (0.96-6.59) | 0.06 |
|  | Grade 3 | 4.31 (1.66-11.15) | 0.003 |
| Nodal status of first BC | |  |  |
|  | Negative | Ref |  |
|  | Positive | 1.18 (0.79-1.77) | 0.42 |
| Size category of first BC | |  |  |
|  | 0-2 cm | Ref |  |
|  | 2-5 cm | 1.31 (0.90-1.91) | 0.16 |
|  | >5 cm | 2.39 (1.26-4.56) | 0.01 |
|  |  |  |  |
|  | CHEK2:CT, no ET | 3.2E+07 (0.00-INF) | 0.99 |
|  | CHEK2:ET, no CT | 1.1E+00 (0.00-INF) | 1.00 |
|  | CHEK2:both CT and ET | 1.3E+07 (0.00-INF) | 0.99 |

Adjusted for age at diagnosis, ER-status, nodal status, size category and grade of primary BC. Abbreviations: CT=chemotherapy; ET=endocrine therapy.

**Table S12**: Output of the full model for the complete-case analysis restricted to patients diagnosed with an ER-positive first breast cancer, with follow-up starting from 5 years after the diagnosis of the first breast cancer, including an interaction term between *CHEK2* c.1100delC status and radiotherapy.

|  | | **>5 years follow-up** | |
| --- | --- | --- | --- |
| No of patients, non-*CHEK2* | | 19,537 | |
| No of CBC events, non-*CHEK2* | | 318 | |
| No of patients, *CHEK2* | | 238 | |
| No of CBC events, *CHEK2* | | 5 | |
|  | |  | |
|  | | HR (95%CI) | P-value |
| *CHEK2* c.1100delC status | | 8.1E-07 (0.00-INF) | 0.99 |
| Systemic therapy | |  |  |
|  | No systemic therapy | Ref |  |
|  | CT, no ET | 0.83 (0.51-1.37) | 0.48 |
|  | ET, no CT | 0.75 (0.50-1.12) | 0.16 |
|  | Both CT and ET | 0.93 (0.61-1.42) | 0.73 |
| Radiotherapy | |  |  |
|  | No radiotherapy | Ref |  |
|  | Radiotherapy | 1.03 (0.77-1.37) | 0.83 |
| Age at diagnosis | | 0.99 (0.98-1.01) | 0.36 |
| Grade of first BC | |  |  |
|  | Grade 1 | Ref |  |
|  | Grade 2 | 0.99 (0.74-1.32) | 0.96 |
|  | Grade 3 | 1.25 (0.90-1.72) | 0.18 |
| Nodal status of first BC | |  |  |
|  | Negative | Ref |  |
|  | Positive | 0.98 (0.74-1.30) | 0.91 |
| Size category of first BC | |  |  |
|  | 0-2 cm | Ref |  |
|  | 2-5 cm | 0.90 (0.70-1.16) | 0.40 |
|  | >5 cm | 0.49 (0.21-1.11) | 0.09 |
|  |  |  |  |
|  | CHEK2:RT | 1.7E+06 (0.00-INF) | 0.99 |

Adjusted for age at diagnosis, nodal status, size category and grade of primary BC. Abbreviations: CT=chemotherapy; ET=endocrine therapy; RT=radiotherapy.

**Table S13**. Multi-state model in all patients: Pooled hazard ratio (HR) estimates, 95% confidence intervals (CI) and p-values for all covariates and all transitions across multiple imputed data analyses.

|  | **Transition 1** |  | **Transition 2** |  | **Transition 3** |  | **Transition 4** |  | **Transition 5** |  | **Transition 6** |  | **Transition 7** |  |
| --- | --- | --- | --- | --- | --- | --- | --- | --- | --- | --- | --- | --- | --- | --- |
| **Number cases** | 82,701 |  | 82,701 |  | 82,701 |  | 82,701 |  | 1,816 |  | 1,816 |  | 1,816 |  |
| **Number events** | 1,816 |  | 7,467 |  | 4,247 |  | 3,548 |  | 281 |  | 124 |  | 94 |  |
|  | **HR (95% CI)** | **P-value** | **HR (95% CI)** | **P-value** | **HR (95% CI)** | **P-value** | **HR (95% CI)** | **P-value** | **HR (95% CI)** | **P-value** | **HR (95% CI)** | **P-value** | **HR (95% CI)** | **P-value** |
| **c.1100delC status** | 2.37 (1.82-3.08) | < 0.001 | 1.30 (1.09-1.56) | 0.003 | 1.00 (0.75-1.34) | 0.98 | 1.07 (0.76-1.49) | 0.70 | 1.23 (0.72-2.10) | 0.46 | 0.60 (0.14-2.52) | 0.49 | 1.21 (0.41-3.53) | 0.73 |
| **Age at diagnosis** | 1.00 (0.99-1.00) | 0.07 | 1.00 (1.00-1.01) | < 0.001 | 1.10 (1.09-1.10) | < 0.001 | 1.05 (1.05-1.05) | < 0.001 | 0.99 (0.98-1.01) | 0.28 | 1.10 (1.07-1.12) | < 0.001 | 1.04 (1.01-1.06) | 0.004 |
| **ER status** |  |  |  |  |  |  |  |  |  |  |  |  |  |  |
| Negative | ref. |  | ref. |  | ref. |  | ref. |  | ref. |  | ref. |  | ref. |  |
| Positive | 0.97 (0.83-1.14) | 0.72 | 0.81 (0.76-0.87) | < 0.001 | 0.90 (0.81-1.00) | 0.04 | 0.97 (0.87-1.07) | 0.51 | 1.21 (0.85-1.72) | 0.29 | 1.00 (0.53-1.86) | 0.99 | 1.10 (0.58-2.09) | 0.77 |
| **Nodal status** |  |  |  |  |  |  |  |  |  |  |  |  |  |  |
| Negative | ref. |  | ref. |  | ref. |  | ref. |  | ref. |  | ref. |  | ref. |  |
| Positive | 1.06 (0.94-1.19) | 0.38 | 2.42 (2.28-2.56) | < 0.001 | 1.30 (1.20-1.40) | < 0.001 | 1.88 (1.74-2.04) | < 0.001 | 1.79 (1.34-2.41) | < 0.001 | 1.05 (0.65-1.71) | 0.84 | 1.62 (0.89-2.94) | 0.11 |
| **Tumor size** |  |  |  |  |  |  |  |  |  |  |  |  |  |  |
| ≤ 2cm | ref. |  | ref. |  | ref. |  | ref. |  | ref. |  | ref. |  | ref. |  |
| 2-5 cm | 1.13 (1.01-1.26) | 0.04 | 1.81 (1.71-1.91) | < 0.001 | 1.22 (1.12-1.32) | < 0.001 | 1.47 (1.35-1.61) | < 0.001 | 1.52 (1.12-2.07) | 0.01 | 1.25 (0.77-2.04) | 0.37 | 1.34 (0.71-2.55) | 0.36 |
| > 5cm | 1.25 (0.98-1.59) | 0.08 | 2.73 (2.48-3.00) | < 0.001 | 1.45 (1.22-1.72) | < 0.001 | 2.23 (1.89-2.65) | < 0.001 | 2.77 (1.70-4.50) | < 0.001 | 0.79 (0.09-7.13) | 0.83 | 1.20 (0.27-5.34) | 0.81 |
| **Tumor grade** |  |  |  |  |  |  |  |  |  |  |  |  |  |  |
| 1 | ref. |  | ref. |  | ref. |  | ref. |  | ref. |  | ref. |  | ref. |  |
| 2 | 1.02 (0.89-1.16) | 0.81 | 1.87 (1.69-2.06) | < 0.001 | 1.09 (1.00-1.18) | 0.05 | 1.24 (1.10-1.38) | < 0.001 | 1.00 (0.66-1.51) | 1.00 | 0.74 (0.43-1.27) | 0.27 | 0.93 (0.44-1.96) | 0.85 |
| 3 | 1.06 (0.91-1.23) | 0.48 | 2.62 (2.36-2.91) | < 0.001 | 1.18 (1.06-1.30) | 0.002 | 1.72 (1.51-1.97) | < 0.001 | 1.28 (0.81-2.01) | 0.28 | 0.98 (0.54-1.80) | 0.96 | 1.00 (0.42-2.36) | 1.00 |
| **Radiation** |  |  |  |  |  |  |  |  |  |  |  |  |  |  |
| No | ref. |  | ref. |  | ref. |  | ref. |  | ref. |  | ref. |  | ref. |  |
| Yes | 1.07 (0.94-1.21) | 0.33 | 1.00 (0.94-1.06) | 0.94 | 0.75 (0.70-0.80) | < 0.001 | 0.78 (0.72-0.84) | < 0.001 | 0.98 (0.70-1.37) | 0.90 | 0.89 (0.56-1.41) | 0.62 | 0.88 (0.43-1.83) | 0.74 |
| **Systemic treatment** |  |  |  |  |  |  |  |  |  |  |  |  |  |  |
| None | ref. |  | ref. |  | ref. |  | ref. |  | ref. |  | ref. |  | ref. |  |
| CT only | 0.77 (0.62-0.96) | 0.02 | 0.98 (0.88-1.09) | 0.69 | 0.86 (0.73-1.01) | 0.07 | 0.87 (0.75-1.00) | 0.06 | 0.84 (0.51-1.36) | 0.47 | 0.89 (0.38-2.08) | 0.78 | 1.12 (0.43-2.89) | 0.82 |
| ET only | 0.70 (0.58-0.83) | < 0.001 | 0.90 (0.81-1.00) | 0.05 | 0.99 (0.88-1.10) | 0.79 | 0.67 (0.58-0.77) | < 0.001 | 1.13 (0.74-1.74) | 0.57 | 0.93 (0.54-1.61) | 0.81 | 0.74 (0.34-1.58) | 0.43 |
| CT+ET | 0.65 (0.55-0.79) | < 0.001 | 0.96 (0.87-1.06) | 0.39 | 0.82 (0.72-0.94) | 0.01 | 0.57 (0.50-0.66) | < 0.001 | 0.92 (0.61-1.39) | 0.69 | 0.86 (0.41-1.79) | 0.69 | 0.60 (0.26-1.40) | 0.24 |

Abbreviations: CT=chemotherapy; ET=endocrine therapy.

**Table S14**. Multi-state model in patients diagnosed with a first ER-positive breast cancer: Pooled hazard ratio (HR) estimates, 95% confidence intervals (CI) and p-values for all covariates and all transitions across multiple imputed data analyses.

|  | **Transition 1** | | **Transition 2** | | **Transition 3** | | **Transition 4** | | **Transition 5** | | | **Transition 6** | | **Transition 7** | |
| --- | --- | --- | --- | --- | --- | --- | --- | --- | --- | --- | --- | --- | --- | --- | --- |
| **Number cases** | 55,175 |  | 55,175 |  | 55,175 |  | 55,175 |  | 1,133 | |  | 1,133 |  | 1,133 |  |
| **Number events** | 1,133 |  | 4,266 |  | 2,817 |  | 2,090 |  | 167 | |  | 80 |  | 55 |  |
|  | **HR (95% CI)** | **P-value** | **HR (95% CI)** | **P-value** | **HR (95% CI)** | **P-value** | **HR (95% CI)** | **P-value** | **HR (95% CI)** | **P-value** | | **HR (95% CI)** | **P-value** | **HR (95% CI)** | **P-value** |
| **c.1100delC status** | 2.55 (1.87-3.48) | < 0.001 | 1.38 (1.12-1.71) | 0.003 | 1.13 (0.81-1.56) | 0.47 | 0.97 (0.63-1.48) | 0.87 | 1.49 (0.79-2.81) | 0.21 | | 0.89 (0.20-4.06) | 0.89 | 0.61 (0.14-2.79) | 0.53 |
| **Age at diagnosis** | 1.00 (0.99-1.00) | 0.38 | 1.00 (1.00-1.01) | 0.01 | 1.10 (1.10-1.10) | < 0.001 | 1.06 (1.05-1.06) | < 0.001 | 1.00 (0.98-1.02) | 0.99 | | 1.09 (1.06-1.12) | < 0.001 | 1.03 (0.99-1.06) | 0.13 |
| **Nodal status** |  |  |  |  |  |  |  |  |  |  | |  |  |  |  |
| Negative | ref. |  | ref. |  | ref. |  | ref. |  | ref. |  | | ref. |  | ref. |  |
| Positive | 1.00 (0.86-1.17) | 0.95 | 2.46 (2.27-2.66) | < 0.001 | 1.31 (1.20-1.44) | < 0.001 | 1.85 (1.66-2.06) | < 0.001 | 1.76 (1.18-2.62) | 0.01 | | 1.55 (0.84-2.87) | 0.16 | 1.76 (0.76-4.07) | 0.19 |
| **Tumor size** |  |  |  |  |  |  |  |  |  |  | |  |  |  |  |
| ≤ 2cm | ref. |  | ref. |  | ref. |  | ref. |  | ref. |  | | ref. |  | ref. |  |
| 2-5 cm | 1.16 (1.01-1.33) | 0.04 | 1.98 (1.85-2.13) | < 0.001 | 1.17 (1.07-1.28) | < 0.001 | 1.48 (1.31-1.67) | < 0.001 | 1.97 (1.35-2.88) | < 0.001 | | 1.01 (0.54-1.90) | 0.97 | 1.13 (0.53-2.40) | 0.75 |
| > 5cm | 1.31 (0.98-1.77) | 0.07 | 2.97 (2.62-3.36) | < 0.001 | 1.50 (1.21-1.85) | < 0.001 | 2.14 (1.72-2.66) | < 0.001 | 3.86 (2.03-7.32) | < 0.001 | | 0.89 (0.10-7.75) | 0.92 | 0.90 (0.09-9.00) | 0.93 |
| **Tumor grade** |  |  |  |  |  |  |  |  |  |  | |  |  |  |  |
| 1 | ref. |  | ref. |  | ref. |  | ref. |  | ref. |  | | ref. |  | ref. |  |
| 2 | 1.04 (0.88-1.22) | 0.67 | 1.87 (1.68-2.09) | < 0.001 | 1.06 (0.96-1.16) | 0.25 | 1.25 (1.10-1.42) | < 0.001 | 0.97 (0.58-1.62) | 0.90 | | 0.65 (0.35-1.20) | 0.17 | 0.94 (0.40-2.22) | 0.90 |
| 3 | 1.06 (0.88-1.29) | 0.54 | 2.99 (2.66-3.37) | < 0.001 | 1.15 (1.02-1.30) | 0.02 | 1.84 (1.59-2.14) | < 0.001 | 1.58 (0.88-2.82) | 0.13 | | 0.70 (0.32-1.54) | 0.38 | 1.01 (0.35-2.91) | 0.98 |
| **Radiation** |  |  |  |  |  |  |  |  |  |  | |  |  |  |  |
| No | ref. |  | ref. |  | ref. |  | ref. |  | ref. |  | | ref. |  | ref. |  |
| Yes | 1.07 (0.92-1.25) | 0.35 | 0.99 (0.91-1.08) | 0.88 | 0.73 (0.67-0.80) | < 0.001 | 0.77 (0.69-0.86) | < 0.001 | 1.16 (0.74-1.82) | 0.53 | | 1.08 (0.60-1.94) | 0.80 | 0.98 (0.41-2.33) | 0.96 |
| **Systemic treatment** |  |  |  |  |  |  |  |  |  |  | |  |  |  |  |
| None | ref. |  | ref. |  | ref. |  | ref. |  | ref. |  | | ref. |  | ref. |  |
| CT only | 0.73 (0.52-1.03) | 0.07 | 0.92 (0.76-1.11) | 0.38 | 0.82 (0.60-1.14) | 0.24 | 0.83 (0.62-1.12) | 0.22 | 0.76 (0.33-1.72) | 0.50 | | 0.92 (0.21-4.05) | 0.91 | 1.24 (0.32-4.75) | 0.75 |
| ET only | 0.66 (0.54-0.81) | < 0.001 | 0.79 (0.68-0.92) | 0.002 | 0.99 (0.87-1.13) | 0.91 | 0.64 (0.52-0.78) | < 0.001 | 1.40 (0.80-2.44) | 0.24 | | 0.77 (0.38-1.53) | 0.45 | 0.76 (0.28-2.06) | 0.58 |
| CT+ET | 0.65 (0.52-0.82) | < 0.001 | 0.84 (0.73-0.97) | 0.02 | 0.84 (0.71-0.98) | 0.03 | 0.58 (0.46-0.71) | < 0.001 | 1.06 (0.60-1.89) | 0.84 | | 0.87 (0.37-2.05) | 0.75 | 0.42 (0.14-1.30) | 0.13 |

Abbreviations: CT=chemotherapy; ET=endocrine therapy.

**Table S15**. Multi-state model in patients diagnosed with a first breast cancer from 2000 onwards: Pooled hazard ratio (HR) estimates, 95% confidence intervals (CI) and p-values for all covariates and all transitions across multiple imputed data analyses.

|  | **Transition 1** | | **Transition 2** | | **Transition 3** | | **Transition 4** | | **Transition 5** | | **Transition 6** | | **Transition 7** | |  |
| --- | --- | --- | --- | --- | --- | --- | --- | --- | --- | --- | --- | --- | --- | --- | --- |
| **Number cases** | 59,296 |  | 59,296 |  | 59,296 |  | 59,296 |  | 910 |  | 910 |  | 910 |  | |
| **Number events** | 910 |  | 3,871 |  | 2,224 |  | 2,510 |  | 107 |  | 51 |  | 42 |  | |
|  | **HR (95% CI)** | **P-value** | **HR (95% CI)** | **P-value** | **HR (95% CI)** | **P-value** | **HR (95% CI)** | **P-value** | **HR (95% CI)** | **P-value** | **HR (95% CI)** | **P-value** | **HR (95% CI)** | **P-value** | |
| **c.1100delC status** | 1.85 (1.16-2.97) | 0.01 | 1.21 (0.92-1.59) | 0.17 | 1.25 (0.81-1.95) | 0.32 | 1.31 (0.90-1.90) | 0.16 | 1.15 (0.34-3.88) | 0.82 | 0.00 (0.00-INF) | 0.96 | 0.00 (0.00-INF) | 0.96 | |
| **Age at diagnosis** | 1.00 (0.99-1.00) | 0.40 | 1.01 (1.01-1.02) | < 0.001 | 1.10 (1.09-1.10) | < 0.001 | 1.05 (1.05-1.05) | < 0.001 | 0.99 (0.97-1.01) | 0.50 | 1.09 (1.05-1.13) | < 0.001 | 1.01 (0.97-1.05) | 0.72 | |
| **ER status** |  |  |  |  |  |  |  |  |  |  |  |  |  |  | |
| Negative | ref. |  | ref. |  | ref. |  | ref. |  | ref. |  | ref. |  | ref. |  | |
| Positive | 0.99 (0.77-1.27) | 0.93 | 0.73 (0.66-0.82) | < 0.001 | 0.85 (0.71-1.01) | 0.07 | 0.84 (0.73-0.95) | 0.01 | 1.49 (0.70-3.14) | 0.30 | 0.56 (0.18-1.77) | 0.32 | 0.89 (0.30-2.66) | 0.84 | |
| **Nodal status** |  |  |  |  |  |  |  |  |  |  |  |  |  |  | |
| Negative | ref. |  | ref. |  | ref. |  | ref. |  | ref. |  | ref. |  | ref. |  | |
| Positive | 1.05 (0.89-1.25) | 0.55 | 2.44 (2.23-2.67) | < 0.001 | 1.28 (1.14-1.43) | < 0.001 | 1.93 (1.74-2.13) | < 0.001 | 2.35 (1.40-3.94) | 0.001 | 1.22 (0.54-2.78) | 0.63 | 2.84 (1.06-7.63) | 0.04 | |
| **Tumor size** |  |  |  |  |  |  |  |  |  |  |  |  |  |  | |
| ≤ 2cm | ref. |  | ref. |  | ref. |  | ref. |  | ref. |  | ref. |  | ref. |  | |
| 2-5 cm | 1.15 (0.98-1.36) | 0.10 | 2.07 (1.92-2.23) | < 0.001 | 1.22 (1.09-1.37) | < 0.001 | 1.52 (1.37-1.69) | < 0.001 | 2.23 (1.30-3.84) | 0.004 | 1.31 (0.58-2.98) | 0.51 | 1.45 (0.51-4.12) | 0.48 | |
| > 5cm | 1.48 (1.10-2.00) | 0.01 | 3.37 (2.99-3.79) | < 0.001 | 1.45 (1.15-1.81) | 0.001 | 2.32 (1.87-2.86) | < 0.001 | 5.23 (2.59-10.56) | < 0.001 | 0.38 (0.03-4.85) | 0.46 | 0.99 (0.08-11.58) | 0.99 | |
| **Tumor grade** |  |  |  |  |  |  |  |  |  |  |  |  |  |  | |
| 1 | ref. |  | ref. |  | ref. |  | ref. |  | ref. |  | ref. |  | ref. |  | |
| 2 | 0.95 (0.78-1.17) | 0.65 | 2.04 (1.76-2.36) | < 0.001 | 1.07 (0.95-1.21) | 0.25 | 1.19 (1.05-1.35) | 0.01 | 1.40 (0.65-3.03) | 0.39 | 0.68 (0.26-1.74) | 0.41 | 0.74 (0.20-2.70) | 0.65 | |
| 3 | 1.05 (0.83-1.33) | 0.67 | 2.98 (2.54-3.49) | < 0.001 | 1.16 (0.99-1.35) | 0.06 | 1.72 (1.48-2.00) | < 0.001 | 2.11 (0.89-5.00) | 0.09 | 0.75 (0.27-2.04) | 0.57 | 0.86 (0.18-4.07) | 0.84 | |
| **Radiation** |  |  |  |  |  |  |  |  |  |  |  |  |  |  | |
| No | ref. |  | ref. |  | ref. |  | ref. |  | ref. |  | ref. |  | ref. |  | |
| Yes | 0.99 (0.83-1.18) | 0.92 | 1.07 (0.98-1.17) | 0.13 | 0.74 (0.67-0.82) | < 0.001 | 0.82 (0.73-0.91) | < 0.001 | 0.93 (0.52-1.65) | 0.79 | 0.62 (0.28-1.39) | 0.25 | 1.25 (0.32-4.94) | 0.74 | |
| **Systemic treatment** |  |  |  |  |  |  |  |  |  |  |  |  |  |  | |
| None | ref. |  | ref. |  | ref. |  | ref. |  | ref. |  | ref. |  | ref. |  | |
| CT only | 0.75 (0.52-1.08) | 0.12 | 1.24 (1.03-1.49) | 0.03 | 0.78 (0.62-0.99) | 0.04 | 0.90 (0.74-1.08) | 0.26 | 0.36 (0.12-1.08) | 0.07 | 1.02 (0.23-4.45) | 0.98 | 0.72 (0.08-6.79) | 0.77 | |
| ET only | 0.69 (0.52-0.91) | 0.01 | 1.02 (0.84-1.25) | 0.81 | 0.93 (0.79-1.10) | 0.38 | 0.82 (0.69-0.98) | 0.03 | 1.06 (0.46-2.43) | 0.90 | 1.58 (0.54-4.60) | 0.40 | 0.80 (0.17-3.88) | 0.78 | |
| CT+ET | 0.67 (0.50-0.90) | 0.01 | 1.21 (1.01-1.45) | 0.04 | 0.77 (0.62-0.95) | 0.02 | 0.62 (0.52-0.75) | < 0.001 | 0.72 (0.31-1.69) | 0.45 | 1.13 (0.24-5.33) | 0.88 | 0.35 (0.06-1.94) | 0.23 | |

Abbreviations: CT=chemotherapy; ET=endocrine therapy.

**Table S16**. Multi-state model in all patients: Hazard ratio (HR) estimates, 95% confidence intervals (CI) and p-values for all covariates and all transitions from the complete-case data analysis.

|  | **Transition 1** |  | **Transition 2** |  | **Transition 3** |  | **Transition 4** |  | **Transition 5** |  | **Transition 6** |  | **Transition 7** |  |
| --- | --- | --- | --- | --- | --- | --- | --- | --- | --- | --- | --- | --- | --- | --- |
| **Number cases** | 29,842 |  | 29,842 |  | 29,842 |  | 29,842 |  | 703 |  | 703 |  | 703 |  |
| **Number events** | 703 |  | 3,098 |  | 1,410 |  | 767 |  | 128 |  | 44 |  | 33 |  |
|  | **HR (95% CI)** | **P-value** | **HR (95% CI)** | **P-value** | **HR (95% CI)** | **P-value** | **HR (95% CI)** | **P-value** | **HR (95% CI)** | **P-value** | **HR (95% CI)** | **P-value** | **HR (95% CI)** | **P-value** |
| **c.1100delC status** | 1.86 (1.17-2.94) | 0.01 | 1.05 (0.79-1.39) | 0.76 | 0.89 (0.53-1.52) | 0.67 | 0.60 (0.25-1.44) | 0.25 | 1.30 (0.57-2.97) | 0.53 | 1.26 (0.15-10.29) | 0.83 | 0.41 (0.05-3.63) | 0.42 |
| **Age at diagnosis** | 0.99 (0.98-1.00) | 0.03 | 1.01 (1.00-1.01) | 0.003 | 1.10 (1.09-1.11) | < 0.001 | 1.04 (1.03-1.05) | < 0.001 | 1.00 (0.98-1.02) | 0.93 | 1.07 (1.03-1.12) | < 0.001 | 1.01 (0.96-1.07) | 0.61 |
| **ER status** |  |  |  |  |  |  |  |  |  |  |  |  |  |  |
| Negative | ref. |  | ref. |  | ref. |  | ref. |  | ref. |  | ref. |  | ref. |  |
| Positive | 0.92 (0.74-1.14) | 0.44 | 0.84 (0.76-0.93) | 0.001 | 0.92 (0.77-1.11) | 0.38 | 0.97 (0.79-1.18) | 0.73 | 1.45 (0.86-2.43) | 0.16 | 0.68 (0.25-1.81) | 0.44 | 2.58 (1.00-6.67) | 0.05 |
| **Nodal status** |  |  |  |  |  |  |  |  |  |  |  |  |  |  |
| Negative | ref. |  | ref. |  | ref. |  | ref. |  | ref. |  | ref. |  | ref. |  |
| Positive | 1.05 (0.88-1.26) | 0.57 | 2.61 (2.40-2.85) | < 0.001 | 1.31 (1.15-1.48) | < 0.001 | 2.02 (1.72-2.37) | < 0.001 | 2.08 (1.34-3.23) | 0.001 | 0.82 (0.37-1.82) | 0.63 | 4.52 (1.61-12.68) | 0.004 |
| **Tumor size** |  |  |  |  |  |  |  |  |  |  |  |  |  |  |
| ≤ 2cm | ref. |  | ref. |  | ref. |  | ref. |  | ref. |  | ref. |  | ref. |  |
| 2-5 cm | 1.08 (0.91-1.27) | 0.38 | 1.76 (1.63-1.90) | < 0.001 | 1.12 (1.00-1.26) | 0.05 | 1.49 (1.27-1.74) | < 0.001 | 1.23 (0.82-1.86) | 0.32 | 0.77 (0.35-1.67) | 0.51 | 0.97 (0.39-2.41) | 0.94 |
| > 5cm | 1.21 (0.84-1.74) | 0.32 | 2.58 (2.25-2.96) | < 0.001 | 1.24 (0.93-1.65) | 0.15 | 1.93 (1.42-2.63) | < 0.001 | 1.90 (0.96-3.78) | 0.07 | 0.00 (0.00-INF) | 0.97 | 1.17 (0.13-10.23) | 0.89 |
| **Tumor grade** |  |  |  |  |  |  |  |  |  |  |  |  |  |  |
| 1 | ref. |  | ref. |  | ref. |  | ref. |  | ref. |  | ref. |  | ref. |  |
| 2 | 1.12 (0.90-1.40) | 0.30 | 1.95 (1.68-2.25) | < 0.001 | 1.03 (0.89-1.18) | 0.73 | 1.41 (1.12-1.79) | 0.004 | 0.96 (0.55-1.69) | 0.90 | 0.35 (0.14-0.87) | 0.02 | 0.76 (0.23-2.46) | 0.64 |
| 3 | 1.23 (0.97-1.57) | 0.08 | 2.68 (2.31-3.12) | < 0.001 | 1.15 (0.97-1.35) | 0.11 | 1.98 (1.53-2.55) | < 0.001 | 1.09 (0.59-2.05) | 0.78 | 0.66 (0.26-1.69) | 0.39 | 0.73 (0.19-2.77) | 0.65 |
| **Radiation** |  |  |  |  |  |  |  |  |  |  |  |  |  |  |
| No | ref. |  | ref. |  | ref. |  | ref. |  | ref. |  | ref. |  | ref. |  |
| Yes | 1.10 (0.90-1.33) | 0.35 | 0.94 (0.86-1.03) | 0.21 | 0.74 (0.66-0.83) | < 0.001 | 0.88 (0.75-1.04) | 0.13 | 0.87 (0.52-1.45) | 0.60 | 0.66 (0.28-1.52) | 0.32 | 0.48 (0.16-1.42) | 0.18 |
| **Systemic treatment** |  |  |  |  |  |  |  |  |  |  |  |  |  |  |
| None | ref. |  | ref. |  | ref. |  | ref. |  | ref. |  | ref. |  | ref. |  |
| CT only | 0.72 (0.54-0.96) | 0.03 | 0.86 (0.74-1.00) | 0.06 | 0.85 (0.66-1.09) | 0.21 | 0.57 (0.44-0.75) | < 0.001 | 0.54 (0.26-1.10) | 0.09 | 1.17 (0.37-3.68) | 0.79 | 0.80 (0.22-2.95) | 0.74 |
| ET only | 0.73 (0.55-0.96) | 0.02 | 0.67 (0.58-0.79) | < 0.001 | 0.97 (0.81-1.16) | 0.72 | 0.56 (0.44-0.71) | < 0.001 | 0.85 (0.42-1.71) | 0.65 | 0.76 (0.26-2.19) | 0.61 | 0.25 (0.05-1.30) | 0.10 |
| CT+ET | 0.72 (0.54-0.96) | 0.03 | 0.76 (0.65-0.89) | < 0.001 | 0.92 (0.73-1.16) | 0.49 | 0.42 (0.32-0.54) | < 0.001 | 0.80 (0.38-1.67) | 0.55 | 1.30 (0.35-4.78) | 0.70 | 0.14 (0.03-0.74) | 0.02 |

Abbreviations: CT=chemotherapy; ET=endocrine therapy.

**Table** **S17**. Multi-state model in patients diagnosed with a first ER-positive breast cancer: Hazard ratio (HR) estimates, 95% confidence intervals (CI) and p-values for all covariates and all transitions from the complete-case data analysis.

|  | **Transition 1** | | **Transition 2** | | **Transition 3** | | **Transition 4** | | **Transition 5** | | **Transition 6** | | **Transition 7** | |
| --- | --- | --- | --- | --- | --- | --- | --- | --- | --- | --- | --- | --- | --- | --- |
| **Number cases** | 23,924 |  | 23,924 |  | 23,924 |  | 23,924 |  | 525 |  | 525 |  | 525 |  |
| **Number events** | 525 |  | 2,180 |  | 1,167 |  | 550 |  | 99 |  | 30 |  | 24 |  |
|  | **HR (95% CI)** | **P-value** | **HR (95% CI)** | **P-value** | **HR (95% CI)** | **P-value** | **HR (95% CI)** | **P-value** | **HR (95% CI)** | **P-value** | **HR (95% CI)** | **P-value** | **HR (95% CI)** | **P-value** |
| **c.1100delC status** | 2.19 (1.36-3.52) | 0.001 | 1.08 (0.78-1.48) | 0.64 | 1.00 (0.56-1.76) | 0.99 | 0.65 (0.24-1.74) | 0.39 | 1.44 (0.62-3.30) | 0.39 | 1.75 (0.20-15.44) | 0.61 | 0.46 (0.05-4.60) | 0.51 |
| **Age at diagnosis** | 0.99 (0.98-1.00) | 0.02 | 1.00 (1.00-1.01) | 0.14 | 1.11 (1.10-1.12) | < 0.001 | 1.05 (1.04-1.06) | < 0.001 | 1.01 (0.99-1.03) | 0.47 | 1.07 (1.02-1.13) | 0.004 | 0.99 (0.93-1.05) | 0.70 |
| **Nodal status** |  |  |  |  |  |  |  |  |  |  |  |  |  |  |
| Negative | ref. |  | ref. |  | ref. |  | ref. |  | ref. |  | ref. |  | ref. |  |
| Positive | 1.03 (0.83-1.28) | 0.80 | 2.46 (2.21-2.74) | < 0.001 | 1.28 (1.11-1.47) | < 0.001 | 1.91 (1.57-2.33) | < 0.001 | 2.02 (1.17-3.46) | 0.01 | 0.83 (0.26-2.62) | 0.74 | 5.69 (1.52-21.27) | 0.01 |
| **Tumor size** |  |  |  |  |  |  |  |  |  |  |  |  |  |  |
| ≤ 2cm | ref. |  | ref. |  | ref. |  | ref. |  | ref. |  | ref. |  | ref. |  |
| 2-5 cm | 1.10 (0.90-1.33) | 0.35 | 1.92 (1.74-2.10) | < 0.001 | 1.13 (0.99-1.29) | 0.06 | 1.48 (1.23-1.78) | < 0.001 | 1.33 (0.83-2.13) | 0.24 | 0.45 (0.14-1.47) | 0.19 | 1.18 (0.43-3.26) | 0.75 |
| > 5cm | 1.15 (0.73-1.81) | 0.54 | 2.80 (2.37-3.31) | < 0.001 | 1.34 (0.98-1.83) | 0.07 | 1.90 (1.30-2.76) | < 0.001 | 2.13 (0.89-5.08) | 0.09 | 0.00 (0.00-INF) | 0.97 | 0.00 (0.00-INF) | 0.98 |
| **Tumor grade** |  |  |  |  |  |  |  |  |  |  |  |  |  |  |
| 1 | ref. |  | ref. |  | ref. |  | ref. |  | ref. |  | ref. |  | ref. |  |
| 2 | 1.12 (0.89-1.41) | 0.35 | 2.02 (1.73-2.37) | < 0.001 | 1.01 (0.87-1.17) | 0.89 | 1.41 (1.10-1.81) | 0.01 | 1.00 (0.52-1.90) | 0.99 | 0.33 (0.11-0.95) | 0.04 | 0.71 (0.18-2.80) | 0.62 |
| 3 | 1.23 (0.95-1.61) | 0.12 | 3.16 (2.68-3.72) | < 0.001 | 1.10 (0.92-1.31) | 0.32 | 2.05 (1.56-2.71) | < 0.001 | 1.52 (0.74-3.12) | 0.26 | 0.32 (0.09-1.14) | 0.08 | 0.80 (0.16-4.10) | 0.79 |
| **Radiation** |  |  |  |  |  |  |  |  |  |  |  |  |  |  |
| No | ref. |  | ref. |  | ref. |  | ref. |  | ref. |  | ref. |  | ref. |  |
| Yes | 1.05 (0.84-1.31) | 0.68 | 1.01 (0.90-1.12) | 0.88 | 0.78 (0.68-0.89) | < 0.001 | 0.89 (0.73-1.08) | 0.23 | 0.82 (0.46-1.45) | 0.49 | 0.97 (0.32-2.93) | 0.95 | 0.46 (0.11-1.84) | 0.27 |
| **Systemic treatment** |  |  |  |  |  |  |  |  |  |  |  |  |  |  |
| None | ref. |  | ref. |  | ref. |  | ref. |  | ref. |  | ref. |  | ref. |  |
| CT only | 0.71 (0.48-1.05) | 0.09 | 0.85 (0.69-1.05) | 0.14 | 0.84 (0.56-1.26) | 0.40 | 0.58 (0.39-0.88) | 0.01 | 0.57 (0.21-1.52) | 0.26 | 2.01 (0.28-14.58) | 0.49 | 0.41 (0.07-2.52) | 0.34 |
| ET only | 0.67 (0.49-0.92) | 0.01 | 0.60 (0.50-0.72) | < 0.001 | 0.94 (0.77-1.16) | 0.59 | 0.65 (0.48-0.86) | 0.003 | 1.15 (0.49-2.69) | 0.74 | 0.56 (0.15-2.11) | 0.39 | 0.62 (0.09-4.10) | 0.62 |
| CT+ET | 0.68 (0.48-0.94) | 0.02 | 0.68 (0.56-0.82) | < 0.001 | 0.98 (0.75-1.27) | 0.86 | 0.50 (0.36-0.69) | < 0.001 | 0.83 (0.32-2.12) | 0.69 | 1.53 (0.31-7.61) | 0.61 | 0.19 (0.03-1.28) | 0.09 |

Abbreviations: CT=chemotherapy; ET=endocrine therapy.

**Table S18.** Multi-state model in patients diagnosed with a first breast cancer from 2000 onwards: Hazard ratio (HR) estimates, 95% confidence intervals (CI) and p-values for all covariates and all transitions from the complete-case analysis.

|  | **Transition 1** | | **Transition 2** | | **Transition 3** | | **Transition 4** | | **Transition 5** | | **Transition 6** | | **Transition 7** | |
| --- | --- | --- | --- | --- | --- | --- | --- | --- | --- | --- | --- | --- | --- | --- |
| **Number cases** | 23,277 |  | 23,277 |  | 23,277 |  | 23,277 |  | 425 |  | 425 |  | 425 |  |
| **Number events** | 425 |  | 1,805 |  | 877 |  | 598 |  | 66 |  | 20 |  | 19 |  |
|  | **HR (95% CI)** | **P-value** | **HR (95% CI)** | **P-value** | **HR (95% CI)** | **P-value** | **HR (95% CI)** | **P-value** | **HR (95% CI)** | **P-value** | **HR (95% CI)** | **P-value** | **HR (95% CI)** | **P-value** |
| **c.1100delC status** | 1.47 (0.72-2.98) | 0.29 | 1.05 (0.69-1.58) | 0.83 | 0.97 (0.43-2.18) | 0.95 | 0.88 (0.36-2.12) | 0.77 | 0.90 (0.20-4.10) | 0.89 | 0.00 (0.00-INF) | 0.98 | 0.00 (0.00-INF) | 0.98 |
| **Age at diagnosis** | 0.99 (0.98-1.00) | 0.13 | 1.01 (1.01-1.02) | < 0.001 | 1.10 (1.09-1.11) | < 0.001 | 1.04 (1.03-1.05) | < 0.001 | 0.99 (0.97-1.02) | 0.69 | 1.05 (0.99-1.11) | 0.14 | 1.00 (0.93-1.08) | 1.00 |
| **ER status** |  |  |  |  |  |  |  |  |  |  |  |  |  |  |
| Negative | ref. |  | ref. |  | ref. |  | ref. |  | ref. |  | ref. |  | ref. |  |
| Positive | 0.87 (0.62-1.23) | 0.44 | 0.88 (0.75-1.04) | 0.13 | 1.00 (0.75-1.32) | 0.97 | 0.84 (0.66-1.07) | 0.16 | 1.57 (0.52-4.71) | 0.42 | 0.25 (0.02-3.82) | 0.32 | 2.20 (0.36-13.44) | 0.39 |
| **Nodal status** |  |  |  |  |  |  |  |  |  |  |  |  |  |  |
| Negative | ref. |  | ref. |  | ref. |  | ref. |  | ref. |  | ref. |  | ref. |  |
| Positive | 1.05 (0.84-1.31) | 0.69 | 2.52 (2.25-2.82) | < 0.001 | 1.28 (1.09-1.50) | 0.002 | 2.13 (1.77-2.55) | < 0.001 | 2.09 (1.11-3.93) | 0.02 | 1.10 (0.37-3.30) | 0.86 | 9.65 (1.44-64.71) | 0.02 |
| **Tumor size** |  |  |  |  |  |  |  |  |  |  |  |  |  |  |
| ≤ 2cm | ref. |  | ref. |  | ref. |  | ref. |  | ref. |  | ref. |  | ref. |  |
| 2-5 cm | 1.09 (0.88-1.35) | 0.44 | 2.00 (1.80-2.23) | < 0.001 | 1.07 (0.92-1.24) | 0.41 | 1.56 (1.31-1.87) | < 0.001 | 2.11 (1.09-4.10) | 0.03 | 0.92 (0.30-2.77) | 0.88 | 0.52 (0.10-2.85) | 0.45 |
| > 5cm | 1.26 (0.82-1.95) | 0.29 | 3.18 (2.69-3.77) | < 0.001 | 0.98 (0.67-1.44) | 0.93 | 2.14 (1.53-2.99) | < 0.001 | 3.86 (1.56-9.56) | 0.004 | 0.00 (0.00-Inf) | 0.99 | 1.39 (0.09-20.93) | 0.81 |
| **Tumor grade** |  |  |  |  |  |  |  |  |  |  |  |  |  |  |
| 1 | ref. |  | ref. |  | ref. |  | ref. |  | ref. |  | ref. |  | ref. |  |
| 2 | 1.11 (0.82-1.49) | 0.50 | 2.32 (1.84-2.92) | < 0.001 | 1.03 (0.86-1.24) | 0.76 | 1.40 (1.07-1.84) | 0.02 | 1.13 (0.41-3.09) | 0.81 | 0.61 (0.13-2.88) | 0.54 | 2.22 (0.17-29.42) | 0.55 |
| 3 | 1.46 (1.05-2.03) | 0.02 | 3.40 (2.69-4.31) | < 0.001 | 1.22 (0.98-1.52) | 0.07 | 2.03 (1.50-2.73) | < 0.001 | 1.57 (0.54-4.58) | 0.41 | 0.92 (0.16-5.40) | 0.93 | 2.03 (0.11-38.48) | 0.64 |
| **Radiation** |  |  |  |  |  |  |  |  |  |  |  |  |  |  |
| No | ref. |  | ref. |  | ref. |  | ref. |  | ref. |  | ref. |  | ref. |  |
| Yes | 0.99 (0.77-1.27) | 0.94 | 0.99 (0.87-1.12) | 0.86 | 0.80 (0.68-0.93) | 0.004 | 0.96 (0.79-1.15) | 0.64 | 0.56 (0.27-1.16) | 0.12 | 0.54 (0.15-1.87) | 0.33 | 0.73 (0.10-5.20) | 0.75 |
| **Systemic treatment** |  |  |  |  |  |  |  |  |  |  |  |  |  |  |
| None | ref. |  | ref. |  | ref. |  | ref. |  | ref. |  | ref. |  | ref. |  |
| CT only | 0.55 (0.35-0.85) | 0.01 | 1.39 (1.04-1.85) | 0.03 | 0.78 (0.55-1.11) | 0.16 | 0.61 (0.43-0.85) | 0.004 | 0.49 (0.11-2.21) | 0.35 | 0.75 (0.11-5.03) | 0.76 | 0.54 (0.02-16.39) | 0.72 |
| ET only | 0.63 (0.44-0.91) | 0.01 | 0.95 (0.72-1.27) | 0.74 | 0.88 (0.68-1.15) | 0.36 | 0.68 (0.51-0.90) | 0.01 | 1.20 (0.37-3.95) | 0.76 | 1.48 (0.16-13.80) | 0.73 | 0.18 (0.01-2.49) | 0.20 |
| CT+ET | 0.67 (0.46-0.98) | 0.04 | 1.26 (0.95-1.67) | 0.10 | 0.83 (0.61-1.13) | 0.23 | 0.45 (0.33-0.62) | < 0.001 | 0.79 (0.24-2.63) | 0.70 | 1.21 (0.09-16.32) | 0.89 | 0.05 (0.00-0.76) | 0.03 |

Abbreviations: CT=chemotherapy; ET=endocrine therapy.
